# Pleiotrophin drives a prometastatic immune niche in breast cancer

**DOI:** 10.1084/jem.20220610

**Published:** 2023-02-24

**Authors:** Debolina Ganguly, Marcel O. Schmidt, Morgan Coleman, Tuong-Vi Cindy Ngo, Noah Sorrelle, Adrian T.A. Dominguez, Gilbert Z. Murimwa, Jason E. Toombs, Cheryl Lewis, Yisheng V. Fang, Fatima Valdes-Mora, David Gallego-Ortega, Anton Wellstein, Rolf A. Brekken

**Affiliations:** 1 Department of Surgery, University of Texas Southwestern Medical Center, Dallas, TX, USA; 2 Hamon Center for Therapeutic Oncology Research, University of Texas Southwestern Medical Center, Dallas, TX, USA; 3 Harold C. Simmons Comprehensive Cancer Center, University of Texas Southwestern Medical Center, Dallas, TX, USA; 4 Cancer Biology Graduate Program, University of Texas Southwestern Medical Center, Dallas, TX, USA; 5 Lombardi Comprehensive Cancer Center, Georgetown University, Washington, DC, USA; 6 Department of Pathology, University of Texas Southwestern Medical Center, Dallas, TX, USA; 7 Cancer Epigenetic Biology and Therapeutics group, Precision Medicine Theme, Children’s Cancer Institute, Sydney, Australia; 8 School of Clinical Medicine, University of NSW Sydney, Sydney, Australia; 9 School of Biomedical Engineering, Faculty of Engineering and Information Technology, University of Technology Sydney, Sydney, Australia; 10 Garvan Institute of Medical Research and The Kinghorn Cancer Centre, Sydney, Australia; 11 School of Clinical Medicine, St Vincent’s Healthcare Clinical Campus, Faculty of Medicine and Health, UNSW Sydney, Sydney, Australia

## Abstract

Metastatic cancer cells adapt to thrive in secondary organs. To investigate metastatic adaptation, we performed transcriptomic analysis of metastatic and non-metastatic murine breast cancer cells. We found that pleiotrophin (PTN), a neurotrophic cytokine, is a metastasis-associated factor that is expressed highly by aggressive breast cancers. Moreover, elevated PTN in plasma correlated significantly with metastasis and reduced survival of breast cancer patients. Mechanistically, we find that PTN activates NF-κB in cancer cells leading to altered cytokine production, subsequent neutrophil recruitment, and an immune suppressive microenvironment. Consequently, inhibition of PTN, pharmacologically or genetically, reduces the accumulation of tumor-associated neutrophils and reverts local immune suppression, resulting in increased T cell activation and attenuated metastasis. Furthermore, inhibition of PTN significantly enhanced the efficacy of immune checkpoint blockade and chemotherapy in reducing metastatic burden in mice. These findings establish PTN as a previously unrecognized driver of a prometastatic immune niche and thus represents a promising therapeutic target for the treatment of metastatic breast cancer.

## Teaser

Metastatic cancer cells thrive in secondary organs by producing PTN and activating the NF-κB pathway. This leads to increased cytokine production, neutrophil recruitment, and T cell dysfunction. Targeting PTN presents a promising addition to the current regimen for treating metastatic TNBC.

## Introduction

Metastasis is a highly inefficient process with several rate-limiting steps (Cameron et al., 2000; Luzzi et al., 1998). As a result, only a small percentage of cancer cells colonize secondary sites and form successful overt metastases. These “metastasis-initiating cells” had to evolve and adapt to overcome the selective pressure of the metastatic cascade (Chambers et al., 2002). Thus, metastatic cancers often demonstrate cellular plasticity (Celià-Terrassa and Kang, 2016) that facilitates evasion of immune surveillance and therapy resistance. Due to enhanced cellular plasticity, metastatic cancer cells can often express developmental proteins that provide survival advantages (Ben-Porath et al., 2008; Lawson et al., 2015). Identifying such key players in the malignant process, especially those that help generate a favorable niche at secondary sites, might reveal novel therapeutic targets. Additionally, developmental proteins have restricted expression and function in healthy adults (Warrington et al., 2000); therefore, these factors are often viable therapeutic targets.

Pleiotrophin (PTN), named due to its diverse functions (Li et al., 1990), is an embryonic neurotrophic factor that binds to glycosaminoglycan chains (Ryan et al., 2021). It is highly expressed in the central nervous system (CNS) during the perinatal period and is downregulated thereafter with low expression in adult organs (Wanaka et al., 1993). Physiological functions of PTN include neuronal development (Landgraf et al., 2008; Yanagisawa et al., 2010), adipocyte differentiation (Gu et al., 2007; Sevillano et al., 2019), osteoclast development (Imai et al., 1998), mammary epithelial cell differentiation (Wellstein et al., 1992), angiogenesis (Papadimitriou et al., 2016), response to injury (Himburg et al., 2010; Yeh et al., 1998), and fetal lung development (Weng et al., 2009; Weng and Liu, 2010). Also, PTN is reported to be expressed in inflammatory diseases such as rheumatoid arthritis (Pufe et al., 2003), atherosclerosis (Li et al., 2010), and experimental autoimmune encephalitis (Liu et al., 1998), suggesting it has immune regulatory functions. RNA expression profiling of normal and tumor tissues has shown PTN to be consistently expressed in glioblastoma (Wang, 2020); however, several studies have indicated PTN is elevated in other cancers such as breast (Ma et al., 2017), lung (Wang and Wang, 2015), and pancreatic cancer (Yao et al., 2017). Even though PTN was discovered in the early 1990s, the function of PTN remains unexplored in metastatic breast cancer with the majority of earlier studies reporting its proangiogenic function in primary tumor development (Papadimitriou et al., 2016).

To study adaptations in metastatic breast cancer, we performed a transcriptomic comparison between highly metastatic and poorly metastatic breast cancer cells. Here, we report PTN to be a “metastasis-associated factor” that is enriched at metastatic lesions. We found that PTN expression is associated with advanced disease in breast cancer patients and that elevated expression of PTN might be an independent predictor of metastasis. Additionally, inhibition of PTN significantly reduced lung metastasis in multiple mouse models of breast cancer. Mechanistically, we found PTN activates the NF-κB pathway in cancer cells, resulting in elevated expression of cytokines including CXCL5. Inhibition of PTN reverts local immune suppression created by neutrophils resulting in increased activation of CD8 T cells. Furthermore, combining anti-PTN therapy with anti–PD-1 and Abraxane additively increased the efficacy of immune checkpoint blockade in mice. Overall, our results provide an in vivo functional analysis of PTN in breast cancer progression and demonstrate that inhibition of this previously understudied protein has significant therapeutic potential for the treatment of metastatic breast cancer.

## Results

### Comparison between highly metastatic and poorly metastatic cells reveals distinct metastasis-associated signature

**Figure 1.**
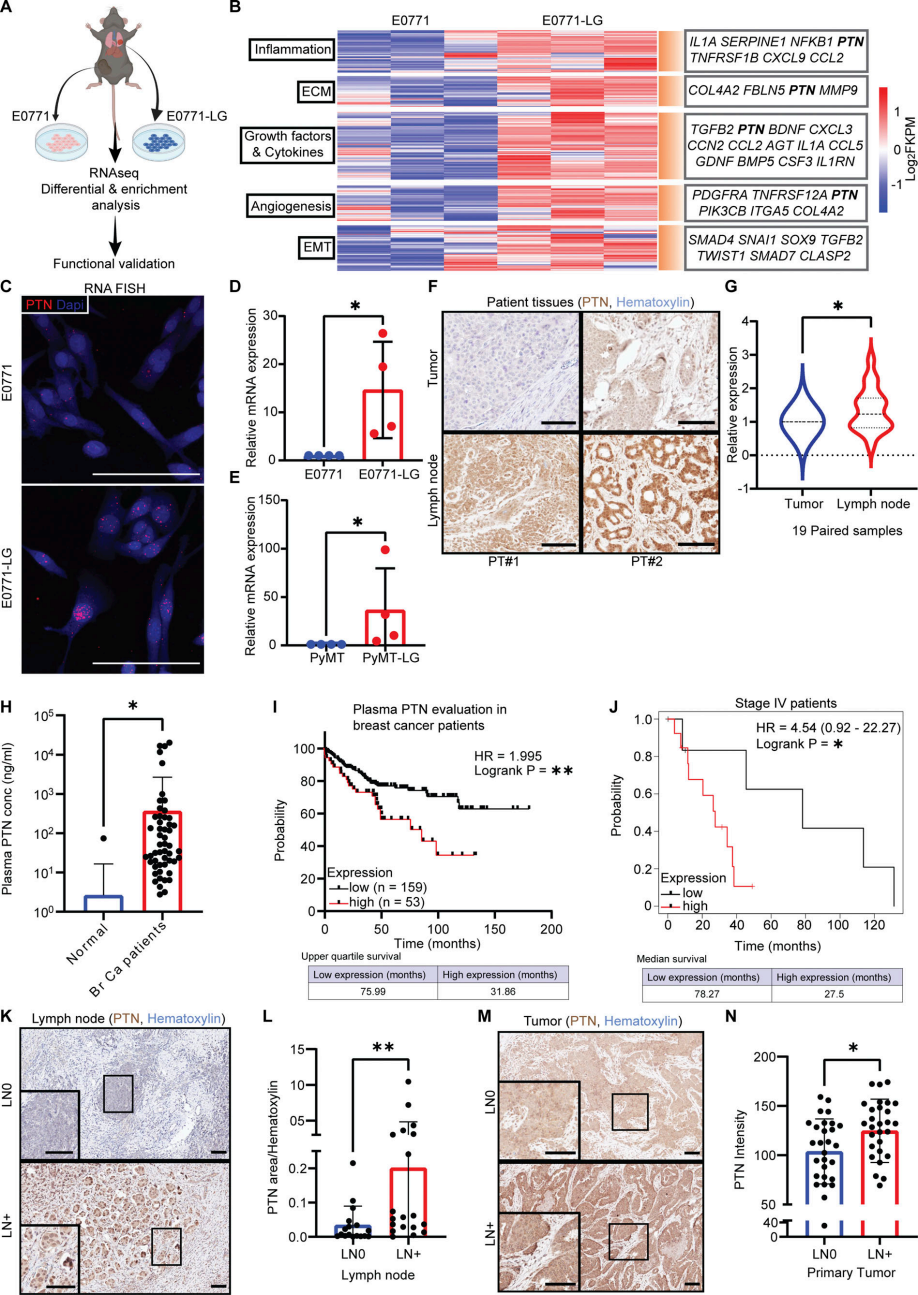
**Metastatic cancer cells have enrichment of neurotropic gene PTN that correlates with poor prognosis in breast cancer patients. (A)** Schematic diagram outlining the strategy for comparison between a highly metastatic lung–derived line, E0771-LG, and a poorly metastatic primary tumor–derived line, E0771. **(B)** Heat map of DEGs between E0771 and E0771-LG cell lines (*n* = 3 each). Relevant genes that are enriched in E0771-LG cells, in each of the important biological processes, are highlighted in the box on the right. **(C)** PTN enrichment in E0771-LG cells was validated by RNA FISH for PTN in cultured cells (*n* = 2). Representative images of two independent replicates are shown at 40× magnification. Scale bar, 100 μm. **(D and E)** PTN enrichment in metastatic lung–derived E0771-LG (D) and PyMT-LG **(**E**)** cancer cells were validated by qPCR. Representative of four independent replicates. Unpaired *t* test; *, P < 0.05. **(F)** 19 pairs of breast cancer patient primary tumor and their corresponding lymph node metastasis were stained for PTN by IHC. Representative images are shown at 20× magnification. Scale bar, 100 μm. **(G)** Quantification of H is shown as violin plot (*n* = 19). Paired *t* test; *, P < 0.05. **(H)** PTN expression in plasma of healthy (no cancer, *n* = 28) individuals and breast cancer patients (*n* = 210) were determined by ELISA and is shown as a bar graph. Unpaired *t* test; *, P < 0.05. Note: Only 1 out of 28 healthy plasma samples had detectable PTN. (**I)** Kaplan–Meier plot for the overall survival of breast cancer patients in H was generated by comparing survival of patients with high (*n* = 53) versus low (*n* = 159) levels of PTN in plasma. Log-rank (Mantel–Cox) test; **, P < 0.01. **(J)** Kaplan–Meier plot derived from TCGA database showing overall survival of stage IV breast cancer patients expressing high (*n* = 13) versus low (*n* = 7) levels of PTN in their primary tumor. Data were generated from https://kmplot.com/analysis/. Log-rank test; *, P < 0.05 **(K)** Lymph node tissue from breast cancer patients were obtained and stained for PTN by IHC. Representative images of PTN staining in lymph nodes from LN^+^ (*n* = 19) and LN^−^ patients (*n* = 17) are shown at 10× magnification. Images at 20× are shown in insets. Scale bar, 100 μm. **(L)** Images from E were quantified in terms of PTN-positive area/hematoxylin area. Unpaired *t* test; **, P < 0.01. **(M)** Primary tumor tissues from LN^+^ (*n* = 29) and LN^−^ (*n* = 29) patients were stained by IHC for PTN. Representative images are shown at 10× magnification and images at 20× are shown in insets. Scale bar, 100 μm. **(N)** Quantitation of G is shown as a bar graph of PTN intensity in primary tumors of LN^−^ and LN^+^ patients. Unpaired *t* test; *, P < 0.05. Tissues and plasma samples (H, K, and M) were obtained from UT Southwestern Tissue Management Shared Resources.

**Figure S1.**
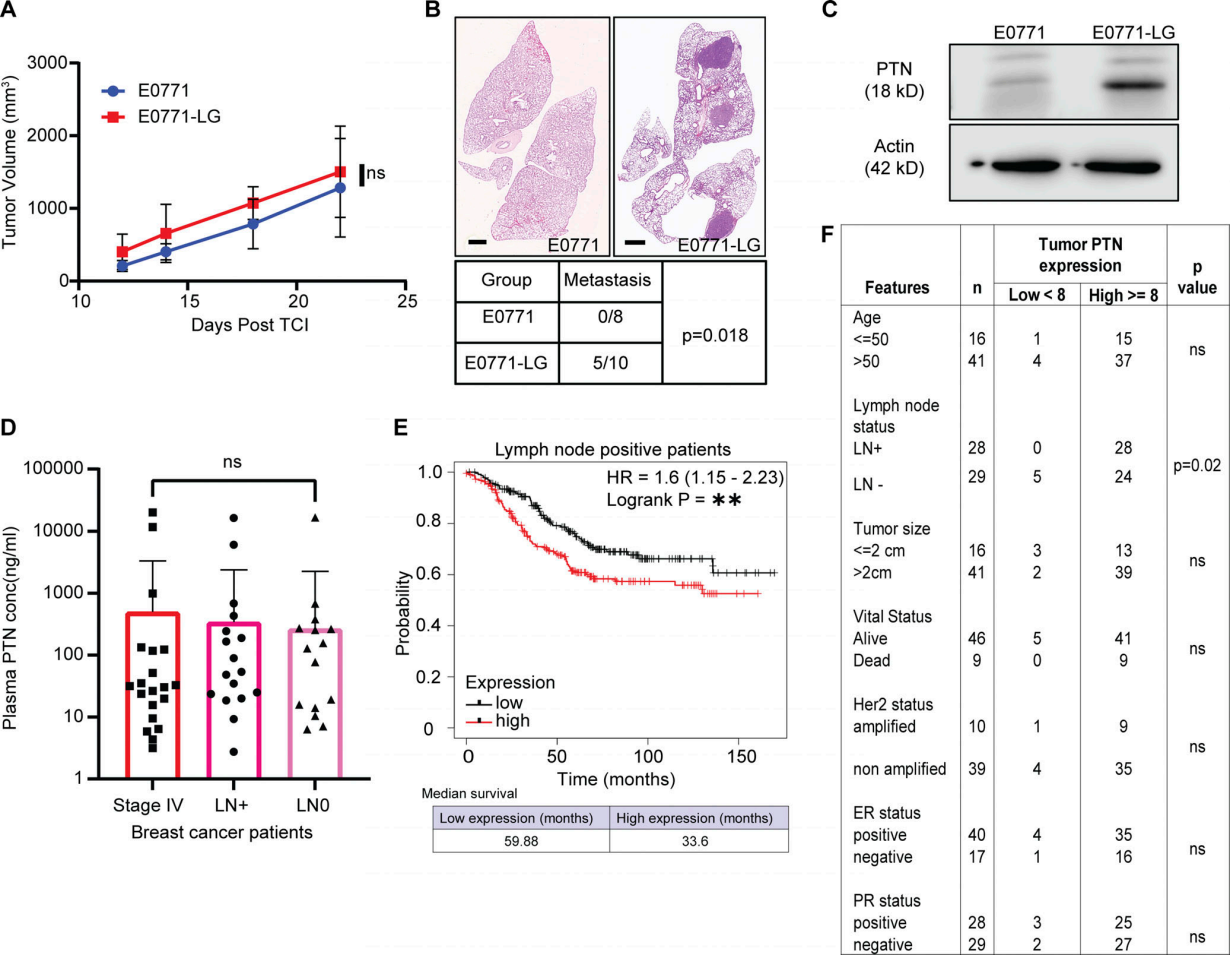
**PTN is associated with metastasis in breast cancer. (A)** Growth curve of orthotopically implanted (fourth mammary fat pad) E0771 parental (*n* = 8) and E0771-LG (*n* = 10) tumors in WT C57BL/6 mice. **(B)** Representative H&E images of lung metastasis from mice bearing E0771 (*n* = 8) or E0771-LG tumors (*n* = 10). Scale bar, 1 mm. Quantification of gross metastasis to lungs in mice bearing E0771 or E0771-LG tumors. Chi-square test; *, P < 0.05. **(C)** Western blot analysis of PTN expression (18 kD) in E0771 and E0771-LG cell lines, representative of three independent replicates. **(D)** Bar graph showing plasma levels of PTN in stage IV (*n* = 67), LN^+^ (*n* = 72), and LN^−^ (*n* = 71) breast cancer patients, as determined by ELISA. Statistical significance was determined using Kruskal–Wallis test. ns > 0.05. **(E)** Kaplan–Meier plot derived from TCGA database showing overall survival of LN^+^ breast cancer patients expressing high (*n* = 220) versus low (*n* = 232) levels of PTN in their primary tumor. Data were generated from https://kmplot.com/analysis/. Log-rank test; **, P < 0.01. **(F)** Primary tumor tissues from Fig. 2 F were scored by a pathologist into PTN-high and -low–expressing tumors. Additional patient history was obtained to determine the correlation between PTN expression and clinical pathophysiological features of patients. Chi-square test; *, P < 0.05. Source data are available for this figure: SourceData FS1.

To understand the pathways involved in the metastatic cascade, we performed RNA sequencing on a pair of syngeneic mouse breast cancer cell lines, E0771 and E0771-LG (Fig. 1 A). E0771 is poorly metastatic and was developed from a spontaneous mouse mammary adenocarcinoma (Ewens et al., 2005; Kitamura et al., 2019; Sugiura and Stock, 1952). E0771-LG cells were derived from a metastatic E0771 lung lesion (Kitamura et al., 2019). E0771 and E0771-LG cells grow similarly as primary tumors (Fig. S1 A), but E0771-LG cells form numerous spontaneous metastatic lung lesions in contrast to parental E0771 cells (Fig. S1 B). Bulk RNA sequencing and downstream differential analysis (Fig. 1 B) allowed us to broadly survey the changes in gene expression involved in various biological processes associated with E0771-LG metastasis. Among the top highly upregulated transcripts in E0771-LG cells, *PTN* was a common gene associated with four out of the five biological gene sets that were different between E0771 and E0771-LG (Fig. 1 B). PTN, being a neurotrophic cytokine, has been studied in the context of neuronal development and has been associated with invasive glioma (Gutmann, 2017; Qin et al., 2017; Shi et al., 2017). Early studies with PTN in breast cancer hinted at potential association of PTN with aggressive disease (Chang et al., 2007). Moreover, midkine (MDK), a protein related to PTN, was recently reported to be associated with aggressive immune evasive melanoma (Cerezo-Wallis et al., 2020; Olmeda et al., 2017), suggesting that these small heparin-binding neurotrophic factors might have unexplored important functions in malignancy. To confirm PTN as a metastasis-associated factor, we compared PTN expression in the syngeneic cell lines, E0771 and E0771-LG, by RNA fluorescence in situ hybridization (RNA-FISH; Fig. 1 C), quantitative PCR (qPCR; Fig. 1 D), and Western blot analysis (Fig. S1 C). PTN was elevated at the RNA level and the protein level in metastatic E0771-LG cells. To further test if PTN expression is associated with metastatic cancer cells, we looked at PTN expression in cells derived from the mouse genetic breast cancer model, *MMTV-PyMT*. Cancer cells isolated from metastatic lungs had higher PTN expression compared with cancer cells isolated from primary tumors (Fig. 1 E). Further, when we compared PTN expression by immunohistochemistry (IHC) in 19 paired tumor and lymph node metastasis samples from human breast cancer patients, we observed higher PTN protein expression in cancer cells in lymph node metastases (Fig. 1, F and G). These data confirm that elevated PTN expression in cancer cells at the metastatic site is conserved across species regardless of the metastatic site, i.e., lungs or lymph nodes.

### High PTN expression is associated with aggressive disease and poor survival in breast cancer patients

PTN is a secreted factor and has been reported to be expressed during embryonic development with limited expression in healthy adults (Sorrelle et al., 2017; Wang, 2020). Therefore, we evaluated PTN expression in breast cancer patient plasma. We tested plasma from 28 healthy individuals (no malignancies) and 210 breast cancer patients by ELISA. PTN was detected at a significantly higher level in breast cancer patients (Fig. 1 H), suggesting it might have potential use as a diagnostic biomarker. Breast cancer patients with plasma PTN concentrations above the detectable level (1 ng/ml) were considered “PTN high” whereas others were considered “PTN low.” Interestingly, even though PTN concentration did not significantly differ between stage IV, lymph node–positive (LN^+^), and lymph node–negative (LN^−^) patients (Fig. S1 D), the PTN-high cohort of patients had a worse overall survival than PTN-low patients (Fig. 1 I). This suggests that changes in PTN level might be a predictor of prognosis in breast cancer patients. Indeed, patients who had high PTN, regardless of disease stage, had a worse outcome clinically. Concurrently, when we plotted overall survival data of stage IV and LN^+^ breast cancer patients from The Cancer Genome Atlas (TCGA), elevated expression of PTN correlated with poor overall survival (Fig. 1 J and Fig. S1 E). PTN IHC staining of lymph nodes and tumor tissue from patients with LN^+^ and LN^−^ disease revealed that PTN expression was significantly higher in lymph nodes (Fig. 1, K and L) as well as in tumors (Fig. 1, M and N) from LN^+^ patients. These slides were confirmed, in a blinded fashion, by a breast pathologist (Y.V. Fang). Further, high PTN was associated with lymph node positivity but not with tumor size, age, or hormone status (Fig. S1 F). This suggests that PTN might be an independent predictor of metastasis and poor outcome.

### Cancer cells and stromal components contribute to overall PTN produced within the tumor microenvironment (TME)

**Figure 2.**
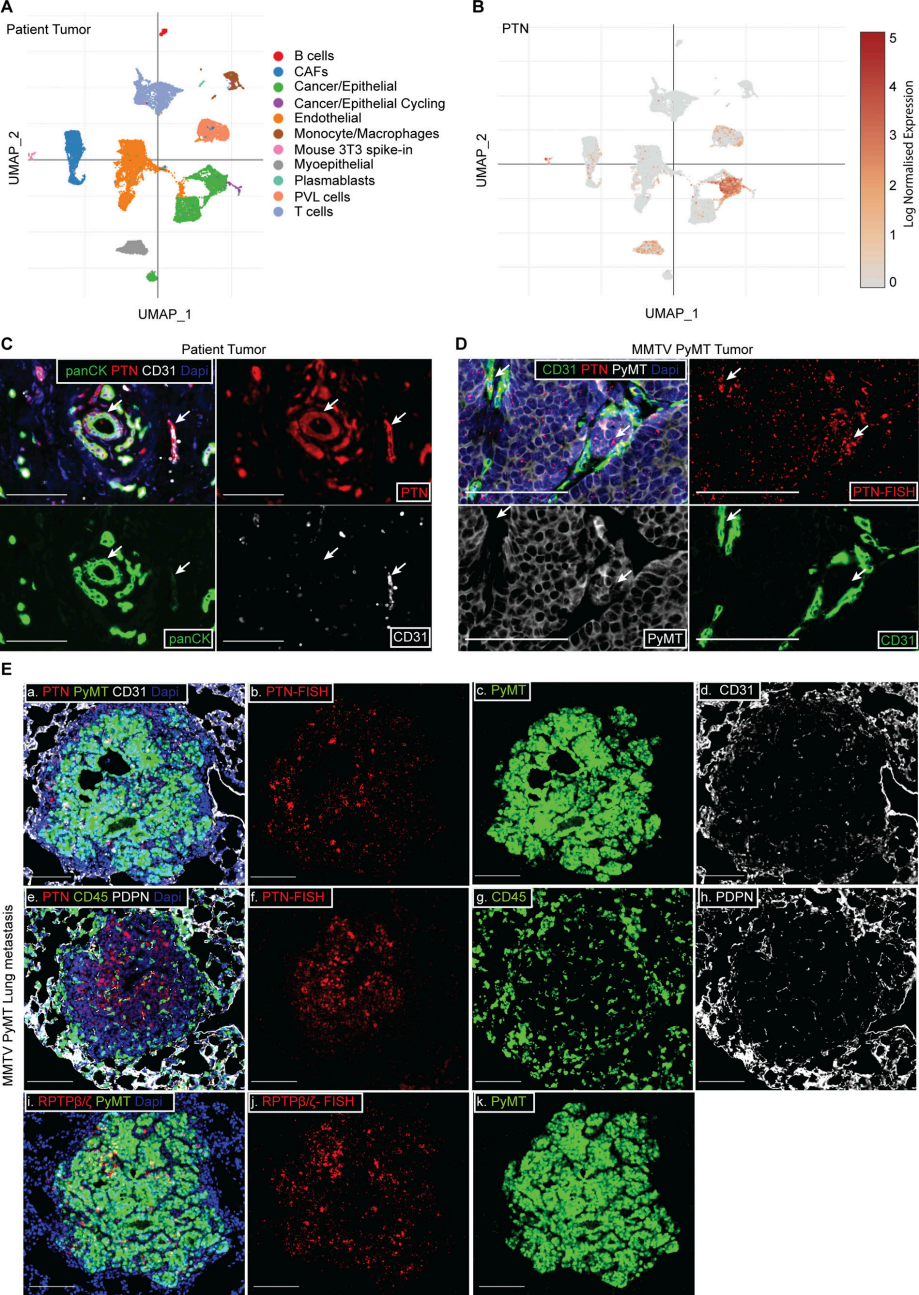
**Cancer cells and endothelial cells produce PTN in the TME. (A)** t-distributed stochastic neighbor embedding (tSNE) plot from scRNAseq of a patient’s primary tumor, BC-P1 CID4471, showing the different cell types within the TME. **(B)** tSNE plot showing the expression of PTN in cell types from A. Data were accessed from the online portal https://singlecell.broadinstitute.org/ (Wu et al., 2021). **(C)** Breast cancer patient primary tumor FFPE sections were stained by multiplex IHC for PTN (red), panCK (cancer cells; green), CD31 (endothelial cells; white), and DAPI (nucleus; blue). Images are representative of two biological replicates. Scale bar, 100 μm. White arrows point at colocalization of the PTN signal with panCK and CD31. **(D)** Representative images of dual RNA FISH of PTN (red) and IHC of CD31 (endothelial cells; green), PyMT (cancer cells; white) in *MMTV-PyMT* primary tumor (*n* = 4). Scale bar, 100 μm. White arrows point at colocalization of the PTN signal with PyMT and CD31. **(E)** Representative images of dual RNA FISH of PTN (red; a, b), PTN receptor RPTPβ/ζ (red; i, j) and IHC of CD31 (white; a, d), PyMT (green; a, c, i, k), CD45 (green; e, g), podoplanin (white; e, h) in *MMTV-PyMT* lung metastases (*n* = 4). Scale bar, 100 μm.

**Figure S2.**
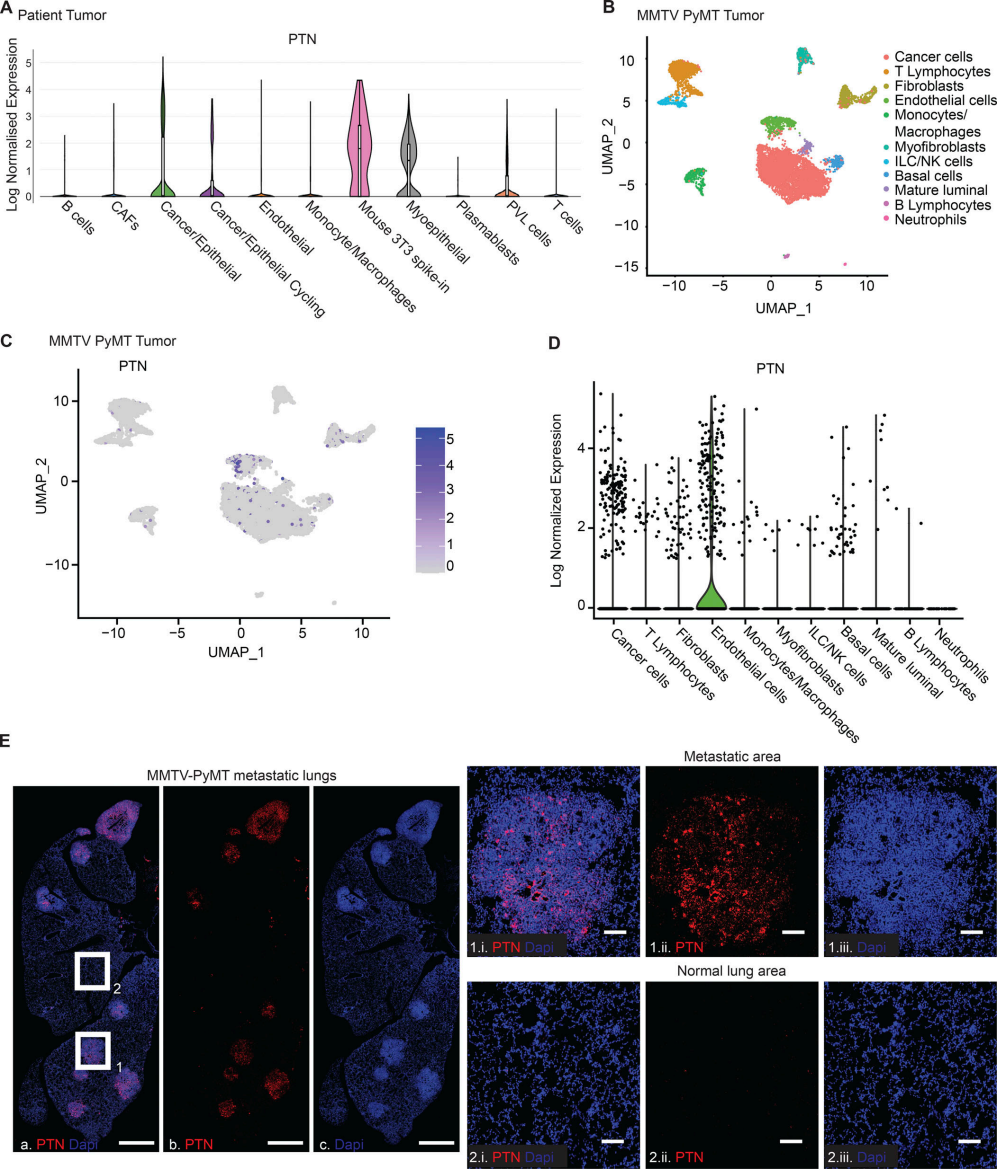
**PTN expression within different cell types of TME. (A)** Violin plot showing PTN expression in different cell types within the TME was generated using breast cancer patient scRNAseq data (BC-P1 CID4471) from a previous study (Wu et al., 2021). **(B)** tSNE plot showing the different cell types identified from scRNAseq of late-stage PyMT tumors. Data accessed from the previous study (Valdés-Mora et al., 2021). **(C)** tSNE plot showing the expression of PTN in different cells from B. **(D)** Violin plot, generated from scRNAseq of MMTV-PyMT primary tumors, showing PTN expression by different cell types within the TME. **(E)** Representative image of RNA FISH of PTN (red) on a lung bearing metastatic lesion from a *MMTV-PyMT* mice (*n* = 4). Scale bar, 1 mm. Boxes 1 and 2 indicate metastatic lesion and normal lung area, respectively. Magnified images at 20× of metastatic lesion and normal lung area are shown in 1.i, 1.ii, 1.iii and 2.i, 2.ii, 2.iii, respectively. Scale bar, 100 μm.

PTN was first purified from MDA-MB-231 cells, a human breast cancer cell line (Wellstein et al., 1992). We also found that PTN is expressed by cancer cells in human breast tumors. However, the level of PTN in stromal cells in the TME is unclear. To address this, we accessed single-cell RNA sequencing data (scRNAseq) from human breast cancer (Wu et al., 2021) and found that PTN was expressed by a population of tumor cells and lowly by multiple stromal components (Fig. 2, A and B; and Fig. S2 A), including endothelial cells. scRNAseq data from late-stage mouse *MMTV-PyMT* tumors also show that PTN is produced by endothelial cells and a subpopulation of cancer cells (Fig S2, B–D). We validated the scRNAseq data in patient and mouse *MMTV-PyMT* tumors by multiplex staining of PTN (by IHC or RNA-FISH) with cancer cell (panCK or PyMT) and endothelial cell (CD31) markers (Fig. 2, C and D). In MMTV-PyMT lungs bearing metastasis, PTN was enriched at the metastatic lesion whereas little or no PTN was detectable in the normal lungs (Fig. S2 E). By combining FISH and multiplex IHC (Fig. 2 E, a–h), we stained metastatic lesions in *MMTV-PyMT* lungs for PTN (RNA-FISH), PyMT antigen (cancer cell marker), CD31 (endothelial marker), CD45 (pan-immune cell marker), and podoplanin (fibroblast, lymphatic maker). Multiplex staining revealed that multiple stromal components can contribute to PTN seen in metastatic lesions; however, the majority of PTN is produced by cancer cells. Furthermore, the most studied receptor for PTN, RPTPβ/ζ (Perez-Pinera et al., 2007; Ryan et al., 2016; Fig. 2 E, i–k), was also enriched in metastatic lung lesions, suggesting PTN signaling at the metastatic site is potentially functionally relevant.

### PTN depletion reduces metastasis in multiple preclinical models of breast cancer

**Figure 3.**
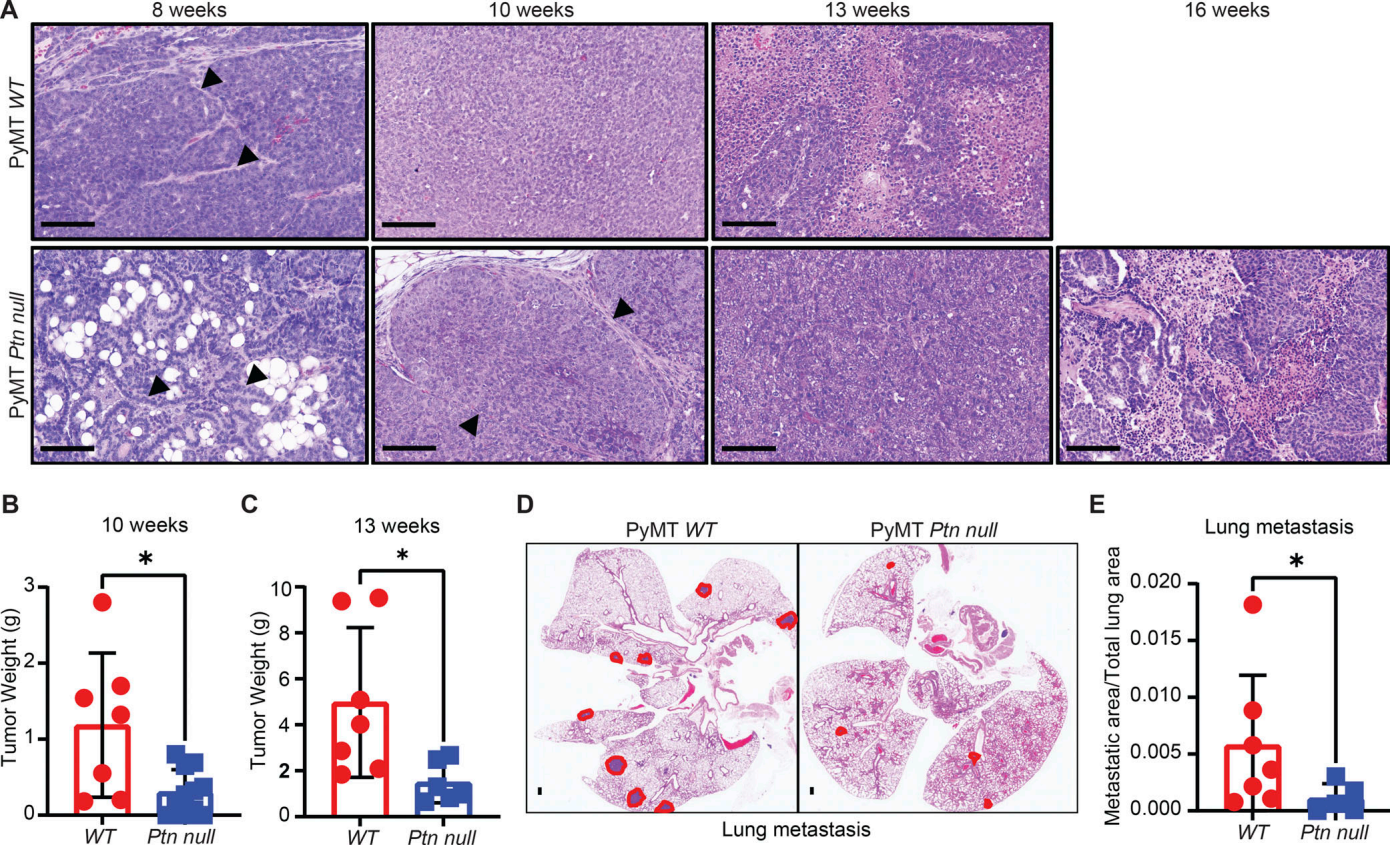
**Genetic ablation of *Ptn* reduces breast cancer progression and metastasis in mice. (A)** Tumor progression in WT and *Ptn null* mice was evaluated by examining tumor histology at 8 (*n* = 4), 10 (*n* = 6), 13 (*n* = 6), and 16 (*n* = 3) wk. Representative H&E images of *MMTV-PyMT* primary tumor from WT or *Ptn null* mice are shown at 20× magnification. Scale bar, 100 μm. Arrowheads in the 8-wk panels point at the myoepithelial layer surrounding intraepithelial neoplasia in WT and at normal mammary epithelial cells in *Ptn null* mammary sections. Arrowheads in the 10-wk panels point at myoepithelial layer surrounding intraepithelial neoplasia in *Ptn null* mammary tumor sections. **(B and C)** Tumor weight in *MMTV-PyMT* WT and *Ptn null* mice at 10 wk (*n* = 7–11; B) and 13 wk (*n* = 6–7; C) of age. Unpaired *t* test; *, P < 0.05. **(D)** Representative H&E images of lung metastasis in *MMTV-PyMT* WT (*n* = 7) and *Ptn null* mice (*n* = 5) at the end of 13 wk. Area of metastasis is circled in red. Scale bar, 100 μm. **(E)** Lung metastatic burden from D was quantitated in terms of metastatic area/total lung area. Unpaired *t* test; *, P < 0.05.

**Figure S3.**
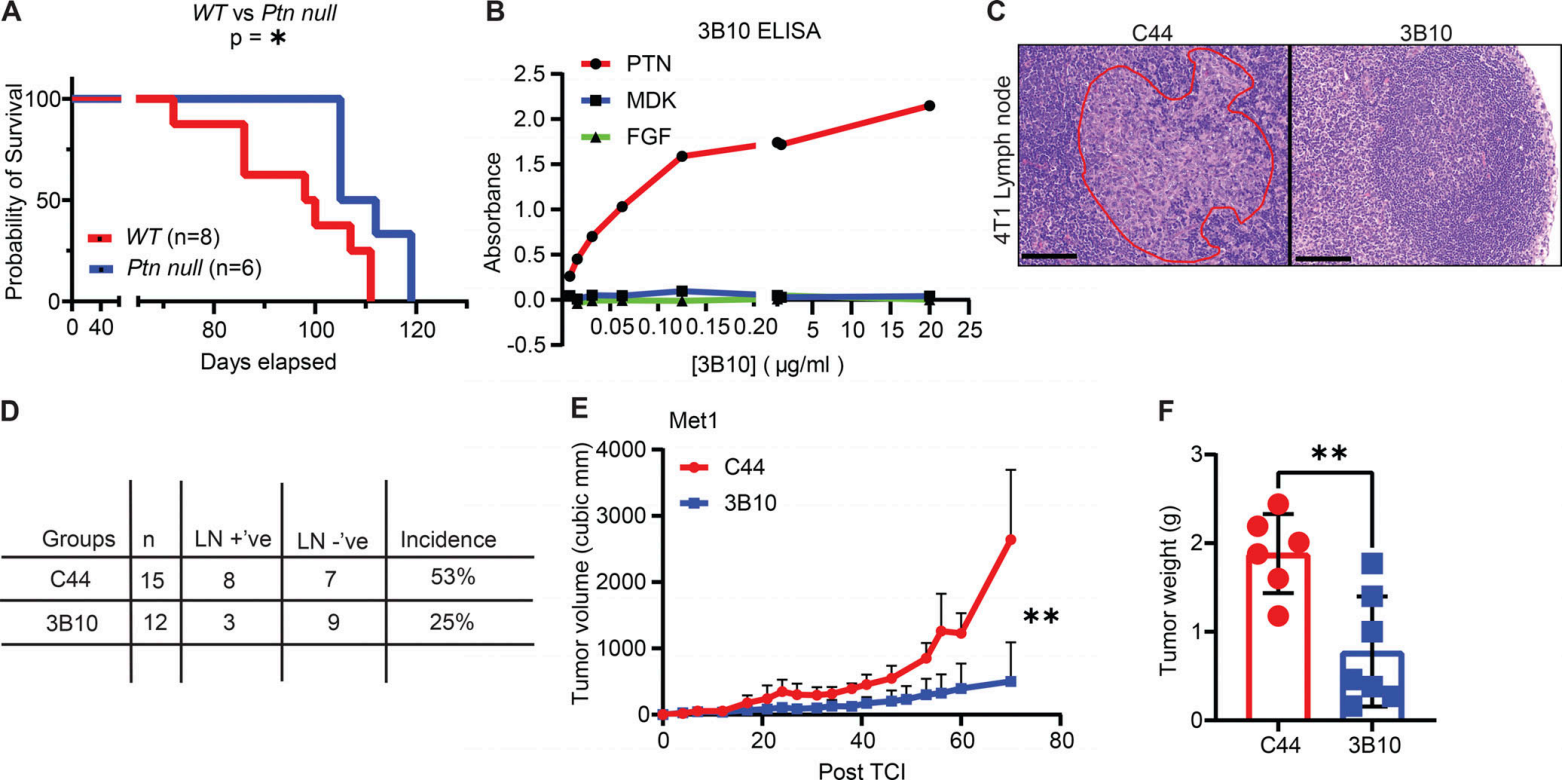
**In vivo inhibition of PTN using a mouse monoclonal antibody, 3B10. (A)** Kaplan–Meier plot comparing the overall survival of *MMTV-PyMT* WT (*n* = 8) versus *Ptn null* mice (*n* = 6). Log-rank (Mantel–Cox) test; *, P < 0.05. **(B)** Specificity of 3B10 for PTN in ELISA. MDK and fibroblast growth factor (FGF) were used as negative controls. Representative of two independent replicates. **(C)** Representation of H&E images of lymph node metastasis in 4T1 tumor–bearing mice treated with either C44 (*n* = 15) or 3B10 (*n* = 12). Area of metastasis are circled in red. Images shown at 20× magnification. Scale bar, 100 μm. **(D)** Quantitation of incidence of metastasis to lymph nodes from C by H&E evaluation. **(E)** Growth curve of orthotopically implanted Met-1 tumors (*MMTV-PyMT* derived) in WT FVB mice treated with IgG control (C44; *n* = 6) or anti-PTN therapy (3B10; *n* = 7). Treatment started once tumors were palpable (∼50 mm^3^). **(F)** Met-1 tumor weight at the end of study in mice. Unpaired *t* test; **, P < 0.01.

Since PTN can be produced by cancer cells and stromal components, we generated *Ptn-null* *MMTV-PyMT* FVB mice to study the function of PTN in breast tumor progression. We compared WT *MMTV-PyMT* and *Ptn-null* *MMTV-PyMT* tumor histology at 8, 10, 13, and 16 wk of age. WT *MMTV*-*PyMT* tumors formed intraepithelial neoplasia by 8 wk, early carcinoma by 10 wk, and late carcinoma with widespread necrosis by 13 wk (Fig. 3 A), similar to previously described studies (Attalla et al., 2021; Lin et al., 2003). *Ptn-null* *MMTV-PyMT* tumors, however, displayed slower progression (Fig. 3 A) showing hyperplasia by 8 wk, intraepithelial neoplasia by 10 wk, early carcinoma by 13 wk, and late carcinoma by 16 wk. This phenotype is complementary to aggressive scirrhous tumors observed in PTN overexpressing *MMTV-PyMT-Ptn* mice reported previously (Chang et al., 2007). Slower tumor progression translated into increased overall survival of *Ptn-null* mice compared with WT *MMTV-PyMT* mice (Fig. S3 A). Alongside, *Ptn-null* mice had smaller tumors at 10 and 13 wk (Fig. 3, B and C) as well as less metastasis to lungs (Fig. 3, D and E) at 13 wk.

**Figure 4.**
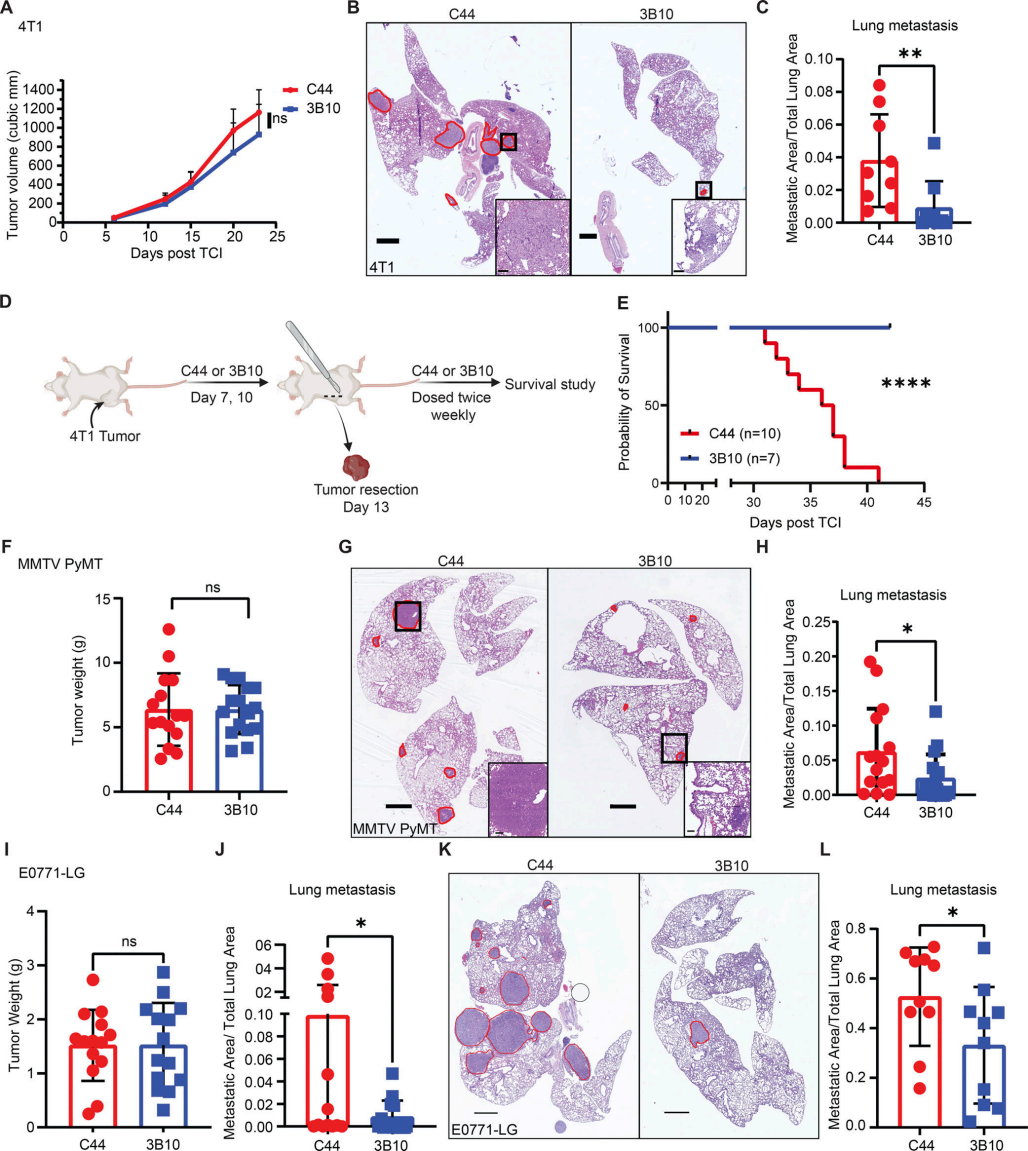
**Pharmacological inhibition of PTN (3B10, mAb) reduces metastasis in multiple preclinical mouse models of breast cancer. (A)** Tumor growth curve of orthotopically implanted 4T1 tumors in WT BALB/c mice treated with IgG control (C44) or anti-PTN therapy (3B10; *n* = 9 mice each). Treatment started once tumors were palpable (∼50 mm^3^). **(B)** Representative H&E images of lung metastasis from 4T1 tumor–bearing mice treated with C44 (*n* = 9) or 3B10 (*n* = 9). Scale bar, 1 mm. Area of metastasis is circled in red. Insets show metastatic lesions in lungs at higher magnification. Scale bar, 100 μm. **(C)** Metastatic area/total lung area from B. Unpaired *t* test; *, P < 0.05. **(D)** Schematic representation of 4T1 resection experiment. **(E)** Kaplan–Meier plot for overall survival of 4T1 tumor–bearing mice from D treated with C44 (*n* = 10) or 3B10 (*n* = 7). Log-rank (Mantel–Cox) test; ****, P < 0.0001. **(F)** Tumor weight in *MMTV-PyMT* mice treated with C44 (*n* = 15) or 3B10 (*n* = 16) at the end of 12 wk of age. Treatment started when mice were 8 wk of age. **(G)** Representative H&E images of lung metastasis from *MMTV-PyMT* mice treated with C44 (*n* = 15) or 3B10 (*n* = 16). Scale bar, 1 mm. Area of metastasis is circled in red. Insets show metastatic lesions in lungs at higher magnification. Scale bar, 100 μm. **(H)** Metastatic area/total lung area from G. Unpaired *t* test; *, P < 0.05. **(I)** Tumor weight of mice bearing E0771-LG tumors treated with C44 or 3B10 (*n* = 14 mice each). Treatment started once tumors were palpable (∼50 mm^3^). **(J)** Metastatic area/total lung area of I. Unpaired *t* test; *, P < 0.05. **(K)** Representative H&E images of metastatic lungs from an experimental metastasis study using E0771LG cells in WT C57BL/6 mice that were treated with C44 or 3B10 (*n* = 10 mice each). Scale bar, 1 mm. Treatment started a day prior to tumor cell injection. Area of metastasis is circled in red. **(L)** Metastatic area/total lung area from K. Unpaired *t* test; *, P < 0.05.

To delineate the functional relevance of PTN in tumor initiation versus tumor progression, we utilized a mouse monoclonal antibody, 3B10, that has been reported to neutralize PTN activity (US patent no. 20140294845A1). We tested the specificity of 3B10 for PTN by ELISA (Fig. S3 B) and confirmed that it only binds to PTN and not to structurally similar protein MDK or other small heparin binding proteins such as fibroblast growth factor. We used 3B10 to determine whether PTN is important for the progression of established breast tumors by exploiting an orthotopic triple-negative breast cancer (TNBC) model, 4T1 (Fig. 4, A–E). Tumor-bearing mice with established and palpable (∼50 mm^3^) tumors were treated with an IgG control antibody, C44, or 3B10. In this aggressive 4T1 model, 3B10 treatment did not change primary tumor growth (Fig. 4 A); however, 3B10 did reduce metastasis to the lungs (Fig. 4, B, C, and J). We also observed more than 50% reduction in the incidence of lymph node metastasis (Fig. S3, C and D). To test whether PTN blockade can improve survival in a clinically relevant setting, we performed a resection study using the 4T1 orthotopic model (Fig. 4, D and E). Tumors were resected when they reached a volume of ∼250 mm^3^. We found all the 3B10 treated mice were alive on the day the last C44 treated mice died, showing a strong survival benefit.

The phenotype of reduction in metastasis after PTN blockade held true in the *MMTV-PyMT* spontaneous breast cancer model as well. In WT *MMTV-PyMT* mice therapy with 3B10 was initiated after 8 wk of age once tumors had already formed. Again, 3B10 treatment did not reduce primary tumor burden (Fig. 4 F) but inhibited metastasis to lungs (Fig. 4, G and H). We also used previously characterized cells derived from *MMTV-PyMT* tumors, Met-1, in an orthotopic setting. Here, 3B10 slowed primary Met-1 tumor growth. We did not see evidence of lung or lymph node metastases in control or 3B10-treated animals bearing Met-1 tumors (Fig. S3, E and F). We further validated the antimetastatic effect in mice bearing E0771-LG orthotopic tumors, where 3B10 again had no effect on primary tumor burden but significantly reduced metastasis to lungs (Fig. 4, I and J).

To determine if PTN is relevant in cancer cell seeding and colonization of secondary sites, we injected E0771-LG cells via the tail vein. 3B10 or C44 therapy was initiated a day prior to tumor cell injection and mice were thereafter dosed twice weekly. 3B10 treatment significantly reduced metastasis (Fig. 4, K and L) suggesting PTN is relevant in the colonization of secondary sites.

### PTN facilitates inflammation, particularly neutrophil recruitment, by activating the NF-κB pathway in cancer cells

**Figure 5.**
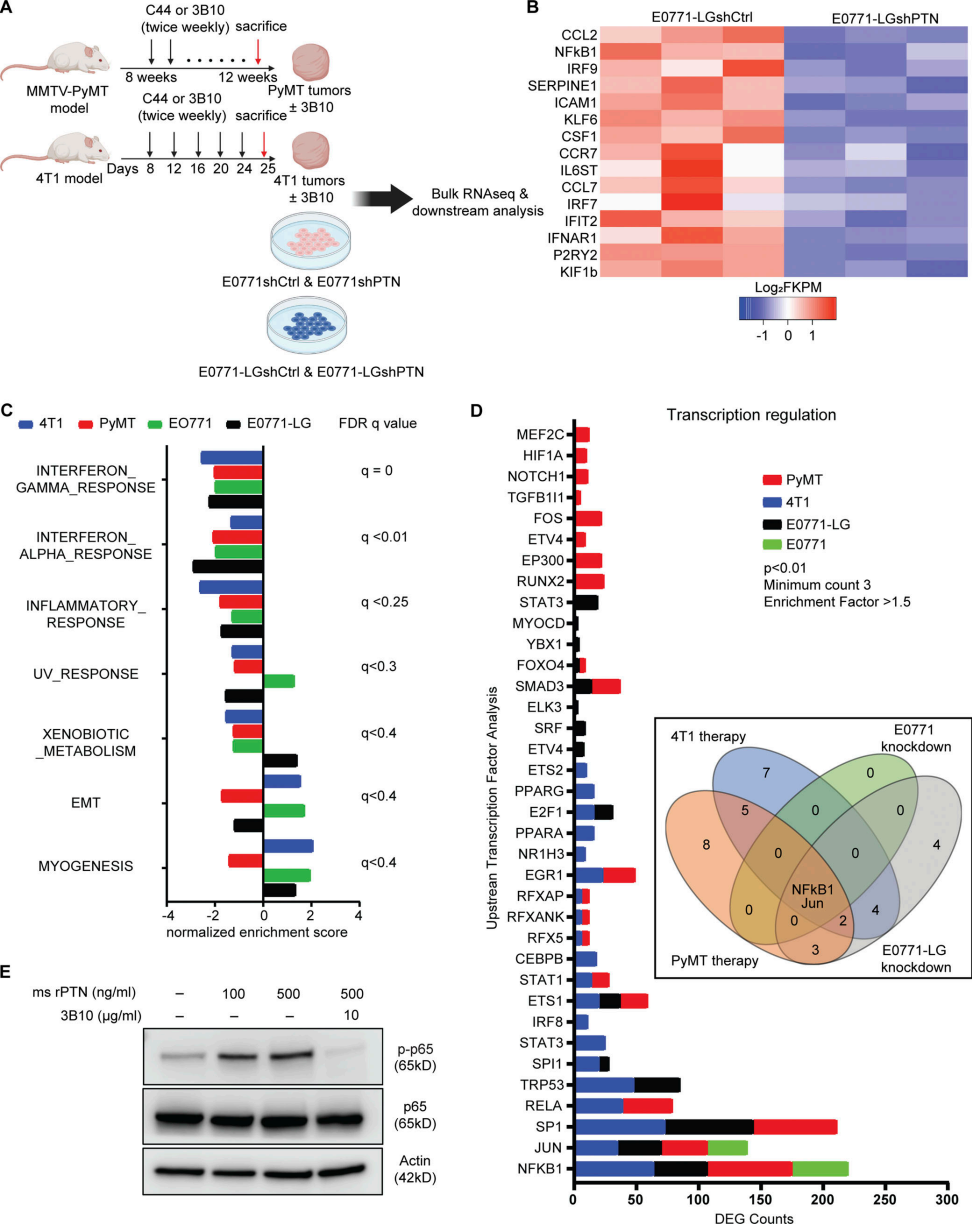
**PTN promotes inflammation by augmenting NF-κB activity in cancer cells. (A)** Schematic outline of the RNA sequencing strategy to investigate the effect of PTN depletion in four different experiments (*n* = 3 each). **(B)** Heat map of differentially expressed inflammation-related genes between E0771-LGshCtrl and E0771-LGshPTN cells (*n* = 3 each). **(C)** Bar graph of normalized enrichment score obtained from gene set enrichment analysis of RNA sequencing data as shown in A. Highest q value amongst the four experiments is shown on the right. **(D)** Bar graph of potential upstream transcription factors regulating DEGs in each of the four experiments in A. Inset displays a Venn diagram showing NF-κB and Jun are the only two common potential transcription factors that can regulate the DEGs from A as determined by TRRUST analysis using metascape. **(E)** Western blot analysis of phospho p65 and total p65 in E0771 cells stimulated with recombinant mouse PTN (100 or 500 ng/ml) in the presence or absence of 3B10 for 30 min. Representative of three independent replicates. Source data are available for this figure: SourceData F5.

**Figure S4.**
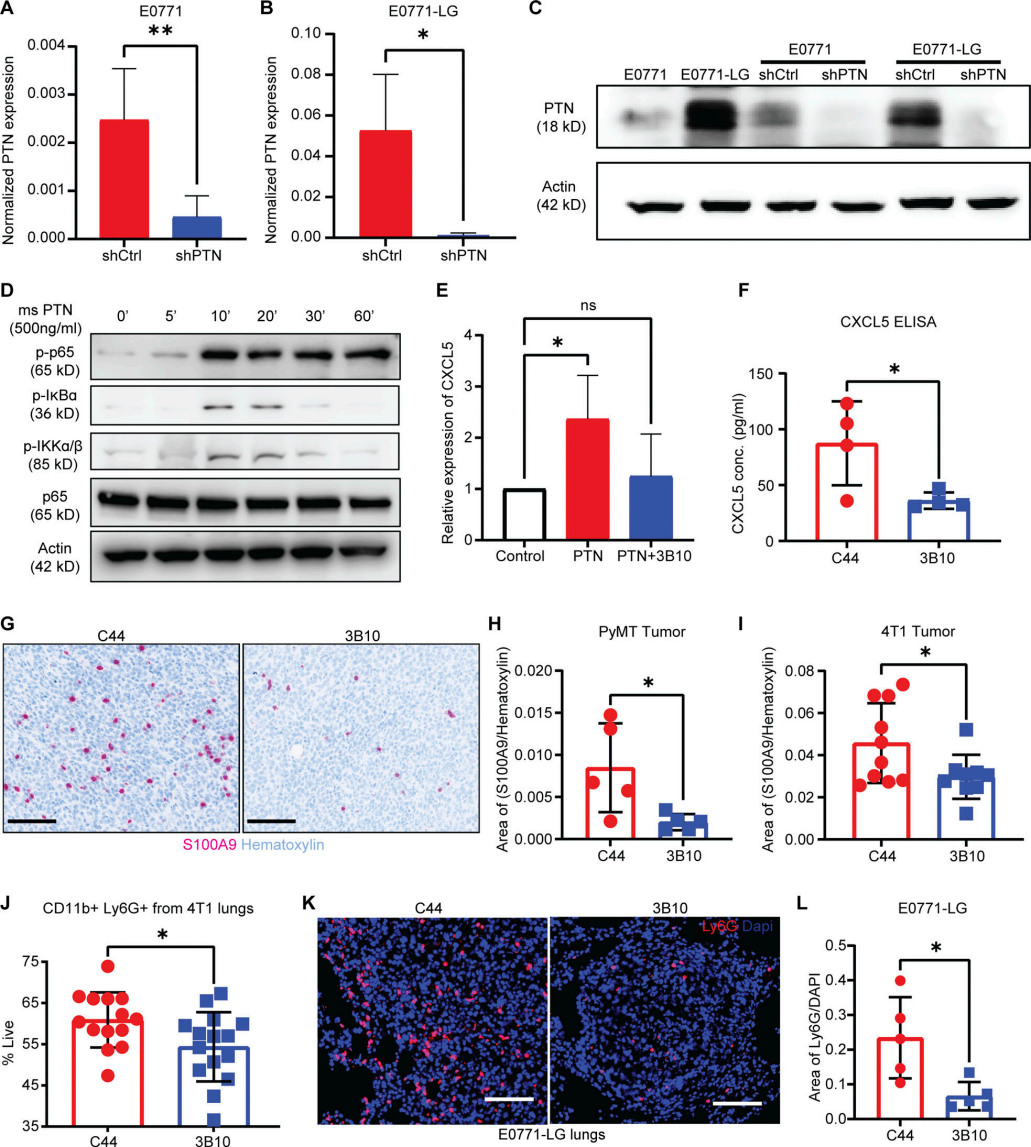
**Validation of knockdown of PTN expression in E0771 and E0771-LG cells. (A)** Normalized PTN expression as determined by qPCR in E0771shCtrl and PTN knockdown cells, E0771shPTN. Unpaired *t* test; **, P < 0.01. Representative of four independent replicates. **(B)** Normalized PTN expression as determined by qPCR in E0771-LGshCtrl and PTN knockdown cells, E0771-LGshPTN. Unpaired *t* test; *, P < 0.05. Representative of three independent replicates. **(C)** Western blot analysis of PTN expression (18 kD) in E0771, E0771-LG, E0771shCtrl, E0771shPTN, E0771-E0771-LGshCtrl, and E0771-LGshPTN cell lines, representative of three independent replicates. **(D)** Western blot analysis of p-p65, p-IκB*α*, p-IKK*α*/β, p-IKKa/B, total p65, and actin in E0771 cells stimulated with recombinant mouse PTN (500 ng/ml) for 0–60 min, representative of two independent replicates. **(E)** Relative expression of CXCL5 mRNA by qPCR upon PTN stimulation (500 ng/ml) in the presence or absence of 3B10 (10 μg/ml). One-way ANOVA; *, P < 0.05, representative of at least three independent replicates. **(F)** CXCL5 expression *MMTV-PyMT* tumors treated with C44 or 3B10 was determined by ELISA. Unpaired *t* test; *, P < 0.05. **(G)** 4T1 (*n* = 9–10) and *MMTV-PyMT* tumors (*n* = 5) from mice treated with C44 or 3B10 were stained for MDSCs (S100A9^+^ cells) by IHC. Representative images are shown at 20× magnification. Scale bar, 100 μm. **(H and I)** Quantification of B. Unpaired *t* test; *, P < 0.05. **(J)** Quantification of neutrophils (CD11b^+^ Ly6G^+^) in 4T1 metastatic lungs from mice treated with C44 (*n* = 14) or 3B10 (*n* = 15) as determined by flow cytometry. Unpaired *t* test; *, P < 0.05. **(K)** E0771-LG metastatic lungs from mice treated with C44 or 3B10 (*n* = 5 each) were stained for neutrophils (Ly6G^+^ cells) by IF. Representative images are shown at 20× magnification. Scale bar, 100 μm. **(L)** Quantification of Ly6G^+^ cells from K. Unpaired *t* tailed test; *, P < 0.05. Source data are available for this figure: SourceData FS4.

**Figure 6.**
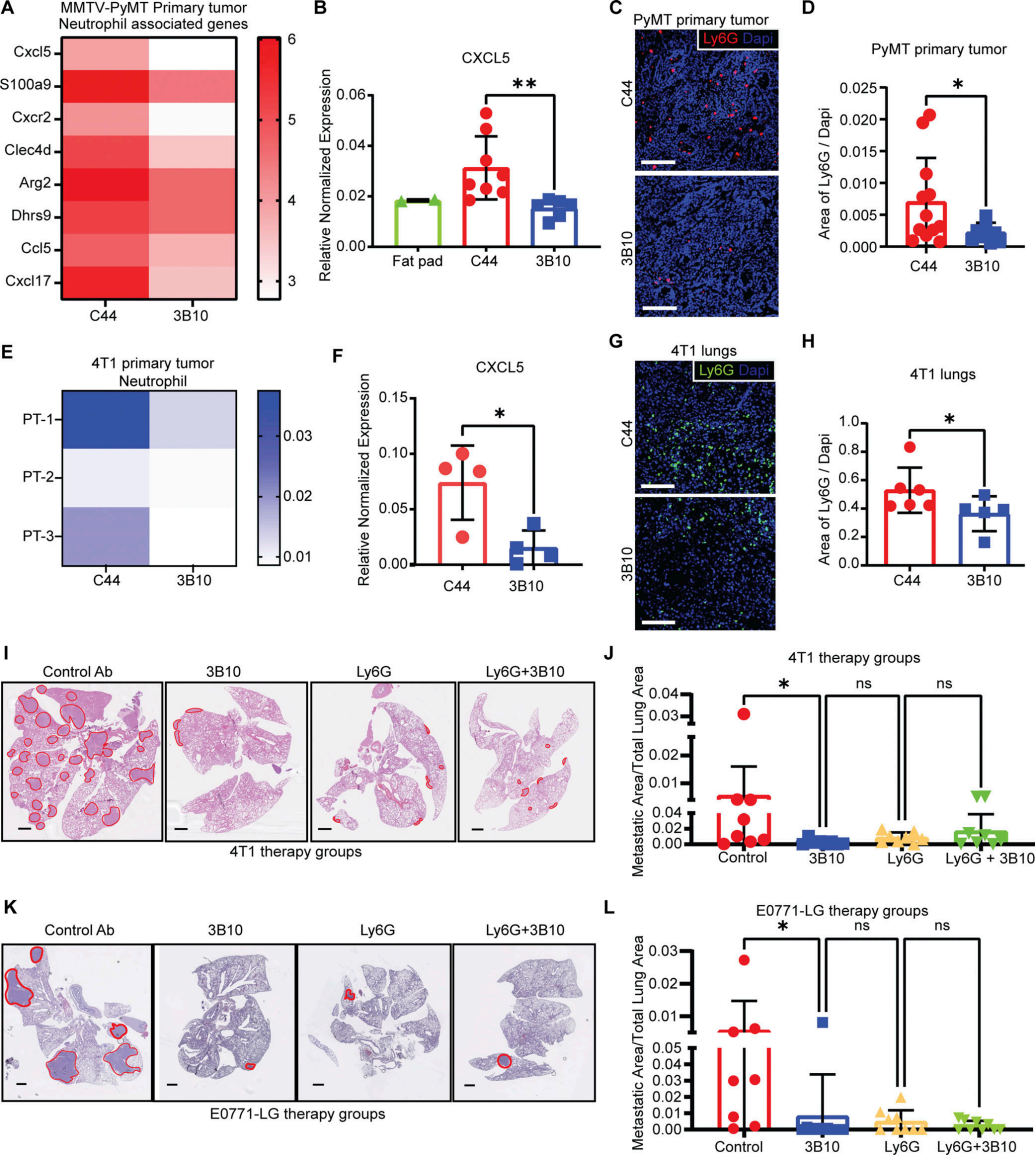
**PTN promotes an inflamed TME by recruiting neutrophils. (A)** Heat map highlighting changes in neutrophil associated genes in *MMTV-PyMT* primary tumors treated with C44 or 3B10 (*n* = 3 each). **(B)** CXCL5 expression in normal mammary fat pad (*n* = 2) or Met-1 (*MMTV-PyMT*–derived) tumors treated with C44 (*n* = 8) or 3B10 (*n* = 6) was determined by cytokine array. Unpaired *t* tailed test; **, P < 0.01. **(C)** *MMTV-PyMT* tumors from mice treated with C44 (*n* = 12) or 3B10 (*n* = 9) were stained for neutrophils (Ly6G^+^ cells) by immunofluorescence (IF). Representative images are shown at 20× magnification. Scale bar, 100 μm. **(D)** Quantification of C. Unpaired *t* tailed test; *, P < 0.05. **(E)** CIBERSORT analysis using RNA sequencing data of 4T1 tumors (*n* = 3 each) shows a decrease in neutrophil infiltration with 3B10 treatment. Analysis was performed at the online portal http://timer.cistrome.org/. **(F)** CXCL5 expression in 4T1 lungs from mice treated with C44 or 3B10 (*n* = 4 each) was determined by cytokine array. Unpaired *t* tailed test; *, P < 0.05. **(G)** 4T1 metastatic lungs from mice treated with either C44 (*n* = 6) or 3B10 (*n* = 5) were stained for neutrophils (Ly6G^+^ cells) by IF. Representative images are shown at 20× magnification. Scale bar, 100 μm. **(H)** Quantification of Ly6G^+^ cells from G. Unpaired *t* tailed test; *, P < 0.05. **(I)** Representative H&E images of lung metastases in WT BALB/c mice bearing 4T1 tumors. Scale bar, 1 mm. WT BALB/c mice were treated with either isotype control (IgG), 3B10, Ly6G, or Ly6G+3B10 (*n* = 8 mice each). **(J)** Quantification of I in terms of metastatic area/total lung area. Kruskal–Wallis test; *, P < 0.05. **(K)** Representative H&E images of lung metastases in WT C57BL/6 mice bearing E0771-LG tumors. WT C57BL/6 mice were treated with isotype control (IgG; *n* = 8), 3B10 (*n* = 10), anti-Ly6G (*n* = 9), or anti-Ly6G+3B10 (*n* = 9). **(L)** Quantification of I in terms of metastatic area/total lung area. Kruskal–Wallis test; *, P < 0.05.

**Figure S5.**
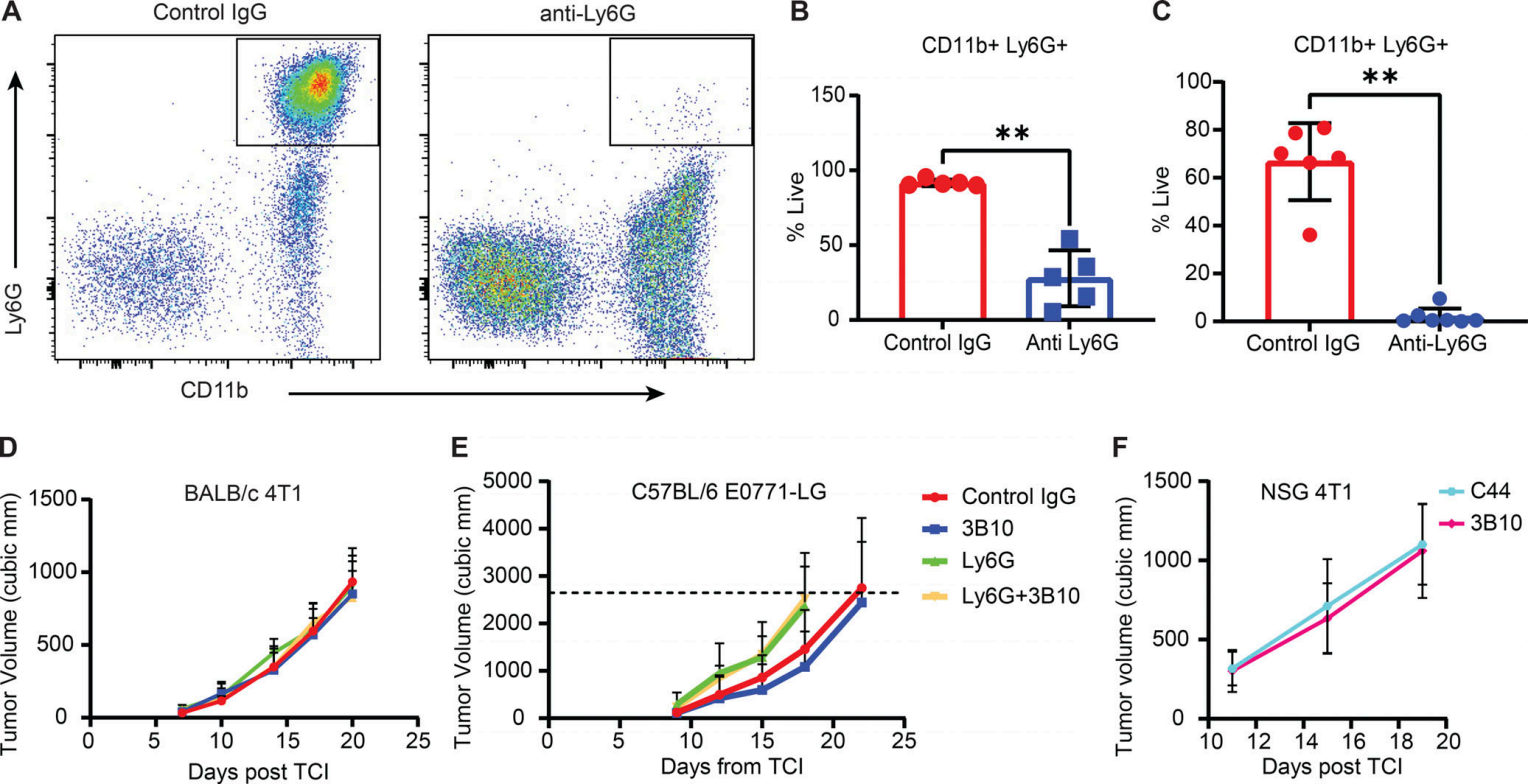
**Effects of PTN inhibition on tumor phenotype. (A)** Confirmation of Ly6G depletion by flow cytometry analysis of CD11b^+^ Ly6G^+^ cells in blood from mice bearing 4T1 (*n* = 5) and E0771-LG (*n* = 6–7) tumor at the end of study. **(B and C)** Quantification of A in 4T1 tumors (B) and E0771-LG tumors (C) respectively. Unpaired *t* test; **, P < 0.01. **(D)** Growth curve of orthotopically implanted 4T1 tumors in WT BALB/c mice treated with IgG control, 3B10, anti-Ly6G, or anti-Ly6G+3B10 (*n* = 8 mice each). **(E)** Growth curve of orthotopically implanted E0771-LG tumors in WT C57BL/6 mice treated with IgG control (*n* = 8), 3B10 (*n* = 10), anti-Ly6G (*n* = 9), or anti-Ly6G+3B10 (*n* = 9). **(F)** Growth curve of orthotopically implanted 4T1 tumors in NSG mice treated with C44 or 3B10 (*n* = 9 mice each).

To uncover pathways that PTN influences to promote metastasis, we took an unbiased approach (Fig. 5 A) through bulk RNA sequencing. 4T1 and *MMTV-PyMT* tumors harvested from mice treated with C44 or 3B10 were subjected to RNA sequencing. Additionally, we generated PTN knockdown E0771 (E0771shPTN; Fig. S4, A and C) and E0771-LG cells (E0771-LGshPTN; Fig. S4, B and C) and performed RNA sequencing from three replicates each. We included E0771-LG, a lung metastasis–derived line, as one of the four RNA sequencing experiments to ensure that only the pathways changing unanimously across primary tumor and lung metastases are included in the analysis. Amongst the differentially expressed genes (DEGs), NF-κB–mediated inflammatory genes (Fig. 5 B), especially cytokines, were downregulated with PTN knockdown or inhibition. We combined gene set enrichment analysis from all four experiments and looked for biological processes that were altered by PTN depletion or blocking. Only interferon and inflammation response-related transcripts (Fig. 5 C) were universally reduced in all scenarios. Further, we then took the top upregulated genes (P < 0.05) in control tumors or cells from each of the four experiments and ran a motif analysis in Metascape. Using the TRRUST algorithm (Han et al., 2018), we determined potential transcription factors that could control DEGs in each of the four experiments. Only two transcription factors were common amongst all, namely NF-κB1 and Jun (Fig. 5 D). To validate PTN-mediated NF-κB activation, we stimulated E0771 cells with recombinant mouse PTN. PTN induced phosphorylation of p65 in a time (Fig. S4 D) and concentration-dependent manner (Fig. 5 E). This effect was abolished in presence of 3B10 (Fig. 5 E). Further, many chemokines that are NF-κB target genes and are important in immune cell trafficking were altered by PTN depletion. We found that alongside activating the various components of the NF-κB pathway (Fig. S4 D), PTN induced the expression of CXCL5 in vitro, an effect that can be abrogated in the presence of 3B10 (Fig. S4 E). Consistently, in our RNA sequencing results, we found that neutrophil-associated genes including CXCL5, a potent neutrophil recruiting cytokine and NF-κB downstream target gene (Chao et al., 2016; Deng et al., 2021), were downregulated with 3B10 treatment (Fig. 6 A). CXCL5 was confirmed to be downregulated at the protein level with 3B10 treatment in MMTV-PyMT–derived tumors (Fig. 6 B and Fig. S4 F). This was accompanied by a reduction in neutrophils (Ly6G^+^ cells) within the TME as determined by IHC (Fig. 6, C and D). These results were validated in the 4T1 model. CIBERSORT analysis of RNA sequencing data in the 4T1 model revealed a reduction of neutrophils in 4T1 tumors from mice treated with 3B10 (Fig. 6 E). Complementing the reduction in Ly6G^+^ cells, we saw a decrease in S100A9^+^ cells, a neutrophil and MDSC marker (Averill et al., 2011), within *MMTV-PyMT* and 4T1 tumors (Fig. S4, G–I). Similar to primary tumors, we observed that 3B10 treatment resulted in a reduction in overall CXCL5 expression in 4T1 metastatic lung lesions (Fig. 6 F). This translated to 3B10 inducing an overall decrease in neutrophils (Ly6G^+^ cells) within lungs in the 4T1 and E0771-LG models, as determined by IHC and flow cytometry (Fig. 6, G and H; and Fig. S4, J–L). Neutrophils have been associated with tumor metastasis in breast cancer (Albrengues et al., 2018; Park et al., 2016; Szczerba et al., 2019; Wu et al., 2020; Yang et al., 2020). To substantiate the data from RNA sequencing, we sought to determine if neutrophils are required for PTN-mediated metastasis. For this, we performed a Ly6G depletion study in two different models (4T1 and E0771-LG) in the presence or absence of 3B10 (Fig. 6, I–L; and Fig. S5, A–E). 3B10 robustly reduced lung metastasis as did the depletion of Ly6G^+^ cells. However, in the absence of Ly6G^+^ cells (Fig. 6, I–L), 3B10 did not have an additive effect, supporting our hypothesis that PTN might be enhancing lung metastasis through neutrophil recruitment.

### Blocking PTN reverts immune suppression and has an additive effect in the treatment of pre-existing metastases when combined with immune checkpoint blockade and chemotherapy

**Figure 7.**
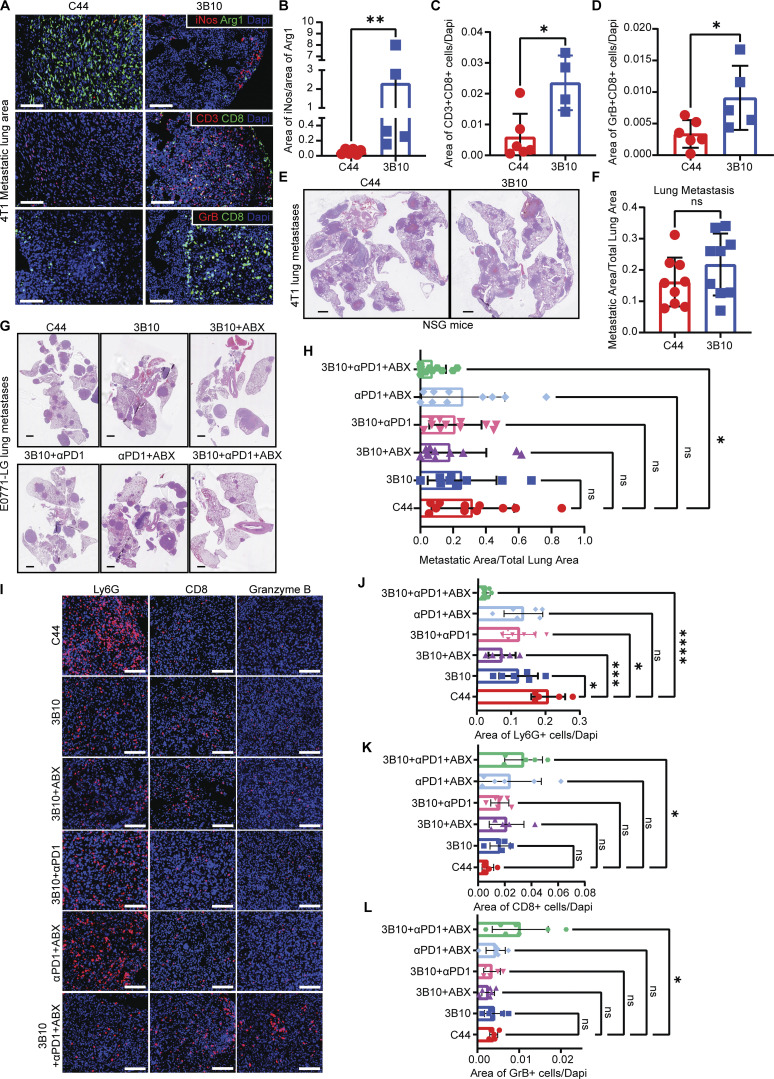
**Inhibition of PTN reverts immune suppression and enhances the effectiveness of immune checkpoint blockade and chemotherapy. (A)** 4T1 metastatic lungs from mice treated with C44 or 3B10 were stained for proinflammatory marker (iNos, red), immunosuppressive marker (Arg1, green), and cytotoxic T cell markers CD3 (red), CD8 (green), and granzyme B (GrB) by IF (*n* = 4–6). Representative images of IF staining of metastatic lesions in 4T1 lungs are shown at 10× magnification. Scale bar, 100 μm. **(B–D)** Quantification of A. Unpaired *t* test; *, P < 0.05; **, P < 0.01. **(E)** Representative H&E images of lung metastases in NSG mice bearing 4T1 tumors. Scale bar, 1 mm. NSG mice were treated with either isotype control C44 (IgG) or 3B10 (*n* = 9 mice each). **(F)** Quantification of M in terms of metastatic area/total lung area. Unpaired *t* test; ns > 0.05. (**G)** Representative H&E images of metastatic lungs from experimental metastasis study using E0771-LG cells in WT C57BL/6 mice that were treated with C44 (*n* = 11), 3B10 (*n* = 9), 3B10+Abraxane (*n* = 11), 3B10+anti-PD-1 (*n* = 11), anti-PD-1+Abraxane (*n* = 10), or 3B10+anti-PD-1+Abraxane (*n* = 11). Scale bar, 1 mm. Treatment started on day 7 after tumor cell injection. **(H)** Quantification of O in terms of gross metastasis. One-way ANOVA test; *, P < 0.05. **(I)** Metastatic lungs from G were stained for neutrophils (Ly6G^+^), CD8 cells, and granzyme B^+^ cells (*n* = 5–7). Representative images of IF staining of metastatic lesions in E0771-LG lungs are shown at 20× magnification. Scale bar, 100 μm. (**J–L)** Quantification of I. One-way ANOVA test; ****, P < 0.0001; ***, P < 0.001; **, P < 0.01; *, P < 0.05.

PTN blockade by 3B10 reduces lung metastasis in multiple models of metastatic breast cancer. Since PTN is enriched in the metastasis microenvironment (Fig. S2 E), we next focused on the changes happening locally in the metastatic lesion. For this, we selected lungs that had metastases from C44 and 3B10 treatment groups and performed multiplex IHC to evaluate the immune landscape. Interestingly, we found that the iNos to Arg1 ratio (Fig. 7, A and B) was higher in the lungs of 3B10 treated mice. Additionally, 3B10 treatment was associated with a higher number of cytotoxic T cells (Fig. 7, A and C; CD3^+^ CD8^+^ cells) and granzyme B^+^ CD8^+^ T cells (Fig. 7, A and D), suggesting an active local antitumor immune response. To determine if the antimetastatic effect of 3B10 was dependent on the adaptive immune system, we implanted 4T1 cells orthotopically in NSG mice and treated with C44 or 3B10. In this setting, 3B10 did not reduce 4T1 lung metastasis (Fig. 7, E and F; and Fig. S5 F). These results suggest that PTN production by cancer cells is critical in maintaining a local immune suppressive metastatic niche to prevent T cell–mediated tumor cell killing.

Pembrolizumab in combination with chemotherapy was recently approved by the Food and Drug Administration for the treatment of metastatic TNBC (https://www.fda.gov/). Given the change in the immune landscape of metastatic lesions after PTN blockade, we speculated that 3B10 combined with immune checkpoint blockade and chemotherapy might show therapeutic efficacy in reducing metastatic lesions. To mimic metastatic TNBC, we injected C57BL/6 female mice with E0771-LG cells via the tail vein and waited for 7 d by which time micrometastases are formed in the lungs (Kitamura et al., 2019). Therapy with IgG control antibody (C44), 3B10, 3B10 plus Abraxane, 3B10 plus anti–PD-1, anti–PD-1 plus Abraxane, or 3B10 in combination with anti–PD-1 and Abraxane was initiated on day 7 after tumor cell injection. Only mice treated with the triple combination therapy of 3B10, anti–PD-1, and Abraxane had significantly reduced lung metastastic burden (Fig. 7, G and H). Additionally, triple therapy was more effective than 3B10 alone or 3B10 plus Abraxane or 3B10 plus anti–PD-1 (Fig. 7, G and H). Additionally, there were significantly lower neutrophils (Ly6G^+^ cells; Fig. 7, I and J), increased CD8^+^ cells (Fig. 7, I and K), and granzyme B^+^ cells (Fig. 7, I and L) in the lungs of mice treated with triple combination therapy. These data support that blocking PTN might be clinically promising when combined with immune checkpoint blockade and chemotherapy for the treatment of metastatic breast cancer.

## Discussion

**Figure 8.**
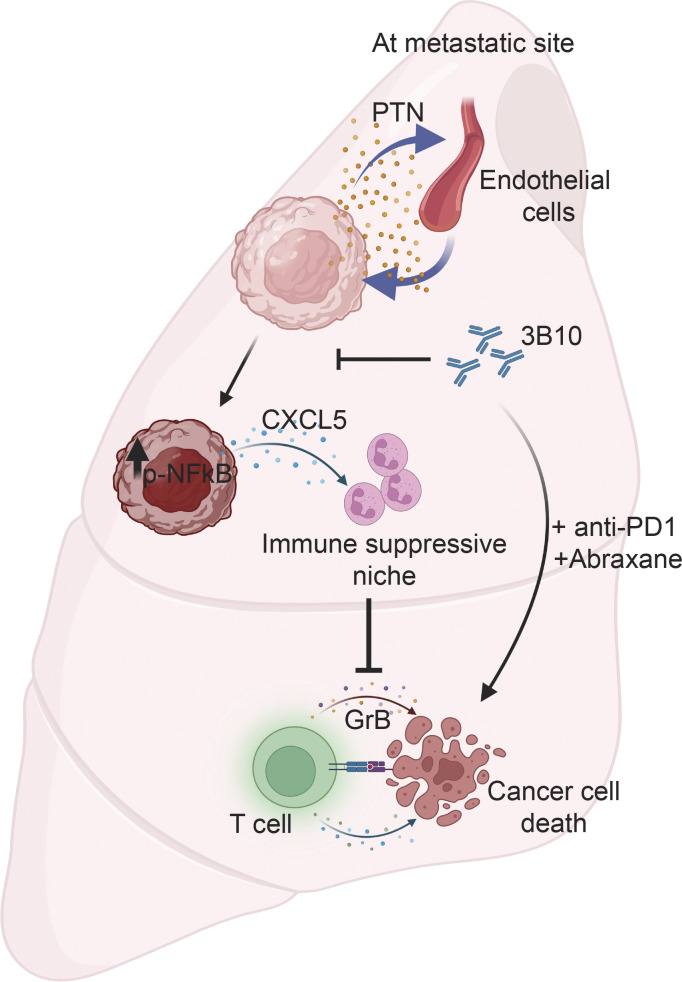
**PTN drives a prometastatic immune niche.** Briefly, the TME of metastatic breast cancer is enriched in the cancer cell and endothelial cell–derived PTN. PTN promotes an inflamed TME driven by NF-κB signaling in cancer cells. PTN-high tumors and lung metastases are rich in immunosuppressive neutrophils that cause T cell dysfunction. Inhibition of PTN reverts immune-suppressive TME thereby increasing the efficacy of checkpoint blockade and chemotherapy in treating pre-existing metastases in mice.

Overall, our results, for the first time, present a comprehensive in vivo functional analysis of PTN (Fig. 8) in breast cancer. PTN in the metastatic microenvironment activates the NF-κB pathway in cancer cells, leading to increased cytokine production including CXCL5, which results in elevated neutrophil recruitment and contributes to immune suppression. Blocking PTN pharmacologically or genetically reduced metastasis by reverting immune suppression created by neutrophils. This leads to increased activation of CD8 T cells locally at the metastatic niche. Combining anti-PTN with anti–PD-1 and Abraxane further reduces lung metastasis, thus presenting a promising addition to the current regimen for treating metastatic TNBC.

PTN abundance and function in the CNS has naturally resulted in it being studied in the context of brain tumors. PTN signaling helps maintain glioblastoma stem cells (Shi et al., 2017) and facilitates glioma cell invasion (Qin et al., 2017). Consequently, RNA expression profiling of normal and tumor tissues in the CNS shows PTN to be consistently expressed at higher levels in glioblastoma (Wang, 2020). PTN expression in other cancers, specifically breast cancer, has provided mixed results (Garver et al., 1994; Giamanco et al., 2017), with data from TCGA showing lower RNA expression of PTN in breast cancer compared with normal tissue (Wang, 2020). However, a previous clinical study on an Asian cohort of patients showed PTN protein expression to be elevated in breast cancer patients (Ma et al., 2017). This is consistent with our results. Our samples included patients from different stages, subtypes, age, and racial background, and represent the largest investigation of PTN in human breast cancer samples to date. A discrepancy between RNA and protein expression levels was also true for a recent study of a PTN family member, MDK, in melanoma (Cerezo-Wallis et al., 2020), thus, highlighting the need to evaluate protein levels for these small growth factors. Notably, even though in our study there were no significant differences in PTN plasma levels between stage IV, LN^+^, and LN^−^ patients, we saw a significant correlation between high PTN expression with metastasis and poor survival. This suggests that regardless of stage, PTN can predict aggressive disease.

When studied in the context of breast cancer, PTN has been reported to be a cancer cell–derived factor only (Wellstein et al., 1992). While cancer cells are the major source of PTN in the TME, we found that multiple stromal cells including endothelial cells also contribute to PTN expression. Though initially studied as a pro-angiogenic factor, 3B10 fails to alter primary tumor growth or microvessel density (data not shown) in TNBC orthotopic mouse models. Instead, we report for the first time, PTN to be a “metastasis-associated factor” in mouse and human breast cancer. Interestingly, in mouse lung metastasis models, PTN expression is enriched at the metastatic niche with little or no expression in surrounding normal lungs. This highlights that metastatic colonies create a local niche to facilitate tumor cell survival (Celià-Terrassa and Kang, 2016). It will be important to investigate what controls local PTN expression. A possibility is that PTN expression is induced as part of an essential adaptation process triggered in cancer cells when they colonize a new site. Aligned with this notion, we report that PTN is essential in forming robust metastasis as blocking it reduces metastatic outgrowth in multiple mouse models of breast cancer. It is intriguing that the absence of PTN slows spontaneous primary tumor growth in *Ptn-null* mice but fails to affect primary tumor growth when 3B10 treatment is initiated once tumors are established. This suggests that PTN might also contribute to early tumor development. This hypothesis is consistent with a previous report of PTN overexpressing *MMTV-PyMT-Ptn* tumors, showing rapid tumor progression compared with *MMTV-PyMT* WT tumors (Chang et al., 2007). Potentially, PTN-related adaptations that occur in cancer cells during early tumor development may be conserved during metastatic colonization events (Nobre et al., 2022). Alternatively, PTN-expressing cancer cells may be better equipped to survive the insults of the metastatic cascade. Consistent with these hypotheses, experimental metastasis assays in the presence of 3B10 reduce metastasis.

Though expressed during injury and in several autoimmune diseases (Sorrelle et al., 2017), PTN function in immune regulation remains unexplored. Through our unbiased transcriptomic approach, we show that blocking or depleting PTN consistently resulted in decreased inflammation. We propose that this is mediated in part by the inhibition of PTN-mediated activation of NF-κB pathway in cancer cells. Previously it was reported that PTN-driven NF-κB activation helps MDA-MB-231 cells escape doxorubicin-induced cell cycle arrest through the expression of CDKN1A (Huang et al., 2018). This was mediated through PTN binding to RPTPβ/ζ and inhibiting its phosphatase activity. Consistently, we found enrichment of RPTPβ/ζ in the metastatic niche, suggesting NF-κB activation could be occurring through this pathway. Given that PTN is promiscuous in its binding to cell surface receptors (Sorrelle et al., 2017), delineating PTN function through each of its receptors presents a challenge in the field. However, our results implicate NF-κB to be a downstream effector of PTN-driven immune regulation. We think that the effect of PTN on neutrophil recruitment is indirect as we were not able to see a phenotypic change in neutrophils in vitro upon direct PTN stimulation (data not shown). Although, given the known function of PTN in maintaining hematopoietic stem cells (Himburg et al., 2012, 2018), it would be interesting to investigate if PTN can bias hematopoietic stem cell differentiation toward the myeloid lineage. Thus far, our data indicate that PTN-stimulated NF-κB activation causes cytokine expression especially CXCL5 in cancer cells, which leads to neutrophil recruitment. It is noteworthy that by inhibiting PTN, the metastatic niche shifted to a proinflammatory local microenvironment with high iNos to Arg1 ratio, increased CD8 T cell infiltration, and increased presence of active effector granzyme B^+^ CD8 cells. Consequently, blocking PTN in combination with anti–PD-1 therapy and Abraxane in mice with established TNBC metastatic disease had a significant therapeutic benefit, especially compared with anti–PD-1 plus Abraxane alone, which is currently being used to treat metastatic TNBC patients. Similar to our findings, PTN family member MDK (Cerezo-Wallis et al., 2020) was associated with immune checkpoint blockade unresponsive melanoma. In that study, MDK promoted immune suppression through NF-κB–driven Arg1 expression in macrophages that mediated T cell dysfunction. These data along with ours present the intriguing concept of blocking PTN and MDK to enhance the efficacy of immune checkpoint blockade.

In summary, our study identifies PTN as a metastasis-associated factor that can facilitate local immune suppression favoring cancer cell colonization at secondary sites and therefore presents a novel therapeutic target in the treatment of metastatic TNBC.

## Materials and methods

### Cells

Murine TNBC cell line, 4T1 (CRL-2539), and immortalized human embryonic kidney cell line, HEK293T (CRL-3216), were obtained from ATCC. MMTV-PyMT (FVB/N) mammary adenocarcinoma-derived cell line, Met-1 (Borowsky et al., 2005), was kindly provided by Dr. Philip Thorpe (UT Southwestern Medical Center, Dallas, TX, USA). Syngeneic murine TNBC (Johnstone et al., 2015) cell line, E0771 (CRL-3461) and E0771-LG (Kitamura et al., 2019), were gifts from Dr. Thorpe (UT Southwestern Medical Center, Dallas, TX, USA) and Dr. Jeffrey Pollard (University of Edinburgh, Edinburgh, UK), respectively. Cells, PyMT and PyMT-LG, were derived from mammary adenocarcinoma and metastatic lungs of a 13-wk-old MMTV-PyMT FVB/N female mice, respectively. For this, tumors and lungs were digested with a cocktail containing collagenase I (45 µm/ml; Worthington), collagenase II (15 µm/ml; Worthington), collagenase III (45 µm/ml; Worthington), collagenase IV (45 µm/ml; Worthington), elastase (0.075 µm/ml; Worthington), hyaluronidase (30 µm/ml; MilliporeSigma), and DNase type I (25 µm/ml; MilliporeSigma) for 1 h at 37°C and passed through a 100-μm cell strainer (Falcon). PyMT and PyMT-LG cells were passaged at least 10 times before use. Mouse hybridoma producing mAb 3B10 (patent no. US 2014/0294845A1) specific for PTN was grown and the supernatant was used to purify 3B10 through standard protein A chromatography. All the above-mentioned cells were cultured in DMEM (Invitrogen) containing 10% FBS and 1% vol/vol penicillin/streptomycin (Thermo Fisher Scientific) at 37°C in a humidified incubator with 5% CO_2_. Cells were confirmed to be free of pathogen before use.

### Animal studies

*MMTV-PyMT* FVB/N, FVB/N, NSG, BALB/c, and C57BL/6 mice were purchased from The Jackson Laboratory. NSG breeder pairs were bred in-house at UT Southwestern. *Ptn-null* C57BL/6 (Muramatsu et al., 2006) mice colony was provided by Dr. John Chute (Cedars Sinai Medical Center, Los Angeles, CA, USA). *Ptn-null* C57BL/6 mice were crossed with WT FVB/N mice to generate *Ptn-null* mice in the FVB/N background. These were further crossed with *MMTV-PyMT* FVB/N males to develop *MMTV-PyMT* *Ptn-null* FVB/N mice. Newly generated mouse colonies were bred for six generations at minimum before use. For in vivo tumor studies, mouse breast cancer cells, 4T1 (1 × 10^5^), E0771 (5 × 10^5^), E0771-LG (5 × 10^5^), and Met-1 (5 × 10^5^) were injected orthotopically into the fourth mammary fat pad of 8-wk-old female BALB/c, C57BL/6, or FVB/N mice. Tumors were measured twice weekly and tumor volumes were calculated using the formula: *V* = (*a* × *b*^2^)/2, where *a* is the length and *b* is the width. Treatments started when tumors were palpable (∼50 mm^3^). Mice were euthanized when tumor volume from the control group reached 2,000 mm^3^. For the survival study in *MMTV-PyMT* background, mice were euthanized when tumors reached a maximum allowable limit. In the 4T1 resection study, mice were given two doses of C44 or 3B10 before resecting tumors (∼250 mm^3^) on day 13. Thereafter, mice were dosed twice weekly and survival was followed. Mice that had primary tumor recurrence were discarded from the study. For experimental metastasis assay, E0771-LG (5 × 10^5^) cells were injected via the tail vein and mice were euthanized on day 19. The endpoint of the experimental metastasis assay was selected based on pilot studies (data not shown). For *MMTV-PyMT* therapy experiments, treatment started at 8 wk of age. For all in vivo studies, mice were randomized into different treatment groups (refer to the figure legend of each figure for the exact therapy window and treatment groups). Treatment dosage consisted of mouse IgG control C44 (15 mg/kg, CRL-1943, twice per week), 3B10 (15 mg/kg, twice per week), anti–PD-1 (5 mg/kg, #BE0273; BioXCell, twice per week), Abraxane (25 mg/kg, #68817-134-50; Celgene, twice per week), anti-rat IgG control (10 mg/kg, #BP0089; BioXCell, every 3 d), and anti-Ly6G (10 mg/kg, BP0075-1; BioXCell, every 3 d). For endpoint experiments, all mice were sacrificed at the same time. Tumors and lungs were weighed and gross metastasis to the lungs was counted. Tissues were fixed in 10% formalin for 48 h or snap-frozen in liquid nitrogen for further studies or digested into single-cell suspension for flow cytometry. All animals were housed in a pathogen-free facility with 24-h access to food and water ad libitum.

All animal procedures in this study were approved by and in compliance with the institutional animal care and use committee policies at UT Southwestern Medical Center at Dallas.

### Clinical sample procurement and inclusion criteria

Lymph node and tumor formalin-fixed paraffin-embedded (FFPE) sections as well as blood plasma from breast cancer patients were obtained from UT Southwestern tissue management shared resources. Three cohorts of patient samples were included in this study: stage IV, LN^+^, and LN^−^ patients. The inclusion criteria for cohort selection were patients with stage IV disease, patients with lymph node metastases of at least 2 mm or larger, and patients with microscopically negative lymph node metastasis, respectively. Case details including overall survival, time of diagnosis, cancer subtype, etc., were also obtained for each patient.

All patient samples and patient information were obtained with voluntary consent from UT Southwestern Tissue Management Shared Resources under an Institutional Review Board protocol approved by the UT Southwestern Institutional Review Board.

### ELISA and cytokine array

Patient blood samples were collected in EDTA tubes. Plasma samples were procured from UT Southwestern tissue management shared resources. These plasma samples were diluted at least 2× or more (as required) and then were used to detect PTN by ELISA using commercial human PTN ELISA kits (EH370RB; Thermo Fisher Scientific). CXCL5 ELISA from tumor lysates was performed using a commercial kit (MX000; R&D Systems) by following their instructions manual.

Mouse cytokine antibody array (abcam, ab133994; RayBiotech, C3AAM-CYT-3-2) was used to detect cytokines from mouse PyMT-derived tumor (Met-1) and 4T1 lung lysates, respectively.

### Survival curve generation

Patients were segregated into PTN-high or PTN-low groups based on plasma PTN concentration. PTN concentrations above the detectable level, 1 ng/ml, were considered PTN high while those lower were considered PTN low. Overall survival data, obtained from UT Southwestern tissue management shared resources, were plotted for PTN high versus PTN low cohort of patients in Graphpad Prism9. Log-rank (Mantel–Cox) test was used to determine significance and hazard ratio. Kaplan–Meier plot for overall survival of stage IV and LN^+^ breast cancer patients based on PTN RNA expression were generated from https://kmplot.com/analysis/.

### IHC

IHC was performed as described (Sorrelle et al., 2019). Briefly, slides were warmed in a 60°C oven for 10 min before deparaffinization and rehydration. Slides were fixed in 10% neutral buffered formalin for 30 min followed by a PBS wash. Antigen retrieval was performed in antigen retrieval buffer (10 mM Tris-HCl, 1 mM EDTA with 10% glycerol) at 110°C for 18 min (∼4–5ψ). Tissue sections were blocked with 2.5% horse serum (S-2012-50; Vector Laboratories) or 2.5% goat serum (S-1012-50; Vector Laboratories) followed by overnight incubation with primary antibody. The next day, slides were washed and incubated with HRP-conjugated secondary antibody for 30 min on a shaker. Slides were then washed three times for 5 min in PBST before developing signal. For developing chromogen signal, Bentazoid 3, 3′ diaminobenzidine (DAB; BDB2004L) was used. Slides were counterstained with hematoxylin and then coverslipped using VectaMount (H-5501; Vector Laboratories) and scanned at 20× using the Hamamatsu NanoZoomer 2.0-HT. For developing fluorescence signal, opal substrates (SKUFP1488001KT, SKU FP1487001KT, SKUFP1497001KT; Akoya Biosciences) were used. When performing multiplex IHC, slides were stripped off of antibodies by incubating in 10 mM citrate buffer (pH 6.2, 10% glycerol) in a pressure cooker at 110°C for 2 min. Sequential staining was performed by stripping after every round of staining before probing for the next antibody. Slides were counterstained with DAPI and then coverslipped using Prolong Gold (#P36931; Life Technologies). Slides were scanned at 40× using the Zeiss Axioscan.Z1 in Whole Brain Microscopy Facility of UT Southwestern.

### Scoring of clinical slides by pathologist

Patient tumor tissues stained for PTN by IHC were scored by a pathologist (Y.V. Fang). Briefly, PTN expression was evaluated by the proportion and intensity of the stained cells. The percentage of the stained tumor cells was categorized as follows: 0 (<5%), 1 (5–25%), 2 (26–50%), 3 (51–75%), and 4 (>75%). The cytoplasmic staining intensity was classified as: 0 (negative), 1 (weak, defined as barely discernable at 4×, visible at 10×), 2 (moderate, defined as visible at 4×), and 3 (strong, dark brown obvious at 4×). The final score was obtained by multiplication of the percentage of stained cells and staining intensity to obtain a total score ranging from 0 to 12. Score of < 8 was considered PTN low while the rest was considered PTN high.

### Antibodies and reagents

For IHC, the following primary antibodies were used: anti-human PTN (1:200, AF252-PB; R&D Systems), anti-PanCK, (1:500, NBP2-29429; Novus Biologicals); anti-human CD31 (1:500, 14-0319-82; Invitrogen), anti-PyMT (1:200, sc53481; Santa Cruz), anti-mouse CD31 (1:500, 77699S; Cell Signaling), anti-podoplanin (1:200, AB11936; abcam), anti-CD45 (1:500, 70257; Cell Signaling), anti-mouse Ly6G (1:200, 551459; BD Biosciences), anti-mouse S100A9 (1:500, 73425; Cell Signaling), anti-CD3e (1:500, PA1-29547; Thermo Fisher Scientific), anti-mouse CD8 (1:1000, 98941S; Cell Signaling), anti-iNos (1:200, PA1-21054; Thermo Fisher Scientific), anti-Arg1 (1:500, 93668S; Cell Signaling), and anti-granzyme B (1:500, 46890S; Cell Signaling). Secondary antibodies used for IHC included: HRP-conjugated secondary anti-goat antibody (MP-7405-50; ImmPRESS; Vector Laboratories), anti-rabbit antibody (MP-7451; ImmPRESS; Vector Laboratories), anti-rat antibody (MP-7404-50; ImmPRESS; Vector Laboratories), or HRP anti-syrian hamster antibody (1:500, 107-035-142; Jackson ImmunoResearch).

For immunoblotting, the following primary antibodies were used: anti-PTN (1:1,000, AF252-PB; R&D Systems), anti-p65 (1:1,000, 8242; Cell Signaling), anti-p-p65 (1:1,000, 3033S; Cell Signaling), anti-actin (1:2,500, A2066; Sigma-Aldrich), anti-p65, anti-p-IκB*α* (1:1,000, 9246; Cell Signaling), and anti–p-IKK*α*/β (1:1,000, 2697S; Cell Signaling). Anti-goat HRP (1:10,000, HRP 705-035-147; Jackson ImmunoResearch) and anti-rabbit HRP (1:10,000, 711-035-152; Jackson ImmunoResearch) were used as secondary antibodies corresponding to the host of primary. Recombinant mouse PTN (6580-PL) was used for in vitro cancer cell stimulation experiments.

### Quantification of IHC

Quantification of IHC was done using Fiji software. DAB intensity was quantified as previously described Nguyen et al. (2013). Briefly, representative images were taken from each section. Each image was color deconvoluted into DAB and hematoxylin. Five random areas were selected to measure the mean gray value. The average mean gray value (f) was obtained, and intensity was calculated using the formula: intensity = 250 − f. To calculate the positively stained area, the deconvoluted DAB image was taken as the threshold at the same intensity value for PTN staining, and the area of the threshold was measured. All calculated areas were normalized using hematoxylin. Quantification represents PTN-positive area/hematoxylin area. For fluorescent images, composite images were created. Single color and co-localized area were calculated by thresholding for each color. All area calculations were normalized to DAPI.

### scRNAseq data collection

Breast cancer patient scRNAseq data (BC-P1 CID4471) was accessed from Wu et al. (2021) using the following online portal: https://singlecell.broadinstitute.org/. Uniform Manifold Approximation and Projection (UMAPs) and violin plots of PTN expression were generated using BC-P1 CID4471 patient data from this study. Mouse *MMTV-PyMT* mammary tumor scRNAseq data was accessed from a previous study (Valdés-Mora et al., 2021). UMAPs and violin plot of PTN expression were generated from MMTV PyMT/WT data set.

### Dual IHC and FISH

FISH on FFPE tissue or fixed cultured cells was performed following the instructions in RNAscope kit (323110; Advanced Cell Diagnostics). PTN (451181), RPTPβ/ζ or PTPRZ1 (460991), positive control (313911), and negative control (310043) probes were purchased from Advanced Cell Diagnostics. When performing dual FISH and IF, following FISH, an HRP blocker (provided in the kit) was used to block further HRP activity. From here on, slides were stained by IHC as detailed in the IHC method section above. For the stripping process, slides were incubated in 10 mM citrate buffer (pH 6.2, 10% glycerol) in a pressure cooker at 110°C for 2 min before probing with the next primary antibody. Slides were counterstained with DAPI and then coverslipped using Prolong Gold (#P36931; Life Technologies). Slides were scanned at 40× using the Zeiss Axioscan.Z1 in Whole Brain Microscopy Facility of UT Southwestern. FISH on fixed cells were scanned at 40× using a laser scanning confocal microscope Zeiss LSM780 at Live Cell Imaging core of UT Southwestern.

### Transfection and knockdown

HEK293T cells were transfected with Lipofectamine 2000 reagent (11668027; Thermo Fisher Scientific) at 70% confluency. The shRNA construct used was PTN KD (TRCN0000071677; Sigma-Aldrich). For a negative control, pLKO.1-puro carrying a sequence targeting EGFP (Sigma-Aldrich) was used. E0771 and E0771-LG target cells were infected with the virus thrice before selection under 3 μg/ml of puromycin. Cells were maintained in puromycin for 6 wk before use.

### Immunoblotting

Cells were lysed in ice-cold radioimmunoprecipitation buffer (50 mM Tris–HCl, pH 8.0, 150 mM NaCl, 0.1% SDS, 0.5% sodium deoxycholate, and 1% Nonidet P-40) for SDS–PAGE. Protein concentration was determined using a BCA Protein Assay Kit (23225; Thermo Fisher Scientific). Samples were boiled for 5 min in Laemmli sample buffer. The proteins were then resolved by SDS–PAGE, transferred to nitrocellulose membranes, and blocked in WestVision (SP-7000-500) block. Membranes were incubated with a primary antibody overnight. The next day, membranes were washed thrice for 5 min with Tris-buffered saline Tween-20 and incubated with horseradish peroxidase-conjugated secondary antibody (Jackson ImmunoResearch Laboratories) for 1 h. Excess secondary antibody was thoroughly removed by washing 3 × 10 min with Tris-buffered saline Tween-20. The membranes were developed with SuperSignal West Pico substrate (34580; Thermo Fisher Scientific). Licor Odyssey Fc imaging system was used for the detection of the immunoreactive bands.

### Flow cytometry

Lungs bearing metastasis were digested with a cocktail containing collagenase I (45 U/ml; Worthington), collagenase II (15 U/ml; Worthington), collagenase III (45 U/ml; Worthington), collagenase IV (45 U/ml; Worthington), elastase (0.075 U/ml; Worthington), hyaluronidase (30 U/ml; MilliporeSigma), and DNase type I (25 U/ml; MilliporeSigma) for 60 min at 37°C and passed through a 70-μm cell strainer (Falcon). Whole blood collected from tumor-bearing mice was spun at 2,000 rpm for 20 min at 4°C to collect the cell pellet. Cells from blood and lungs were washed with PBS and stained with Ghost Viability Dye 510 (BD Biosciences) for 15 min. Cells were then washed and stained with antibodies against CD11b (557657; BD Biosciences) and Ly-6G (740953; BD Biosciences) for 1 h at 4°C. Cells were analyzed using FACS LSRFortessa SORP (BD Biosciences) with the help of the Moody Foundation flow cytometry facility at UT Southwestern Medical Center. Analysis was performed using FlowJo software.

### Tissue histology

Tissues were fixed in 10% neutral buffered formalin for 48 h, embedded in paraffin blocks, and cut into 5-μm sections. H&E was performed and the slides were scanned using at 20× using Hamamatsu NanoZoomer 2.0-HT at Whole Brain Microscopy Facility of UT Southwestern. Lung H&Es were evaluated for metastasis. Metastasis was quantitated as the area of metastasis/total lung area. Areas were determined using NDPview software.

### RNA isolation and qPCR

RNA was isolated from cells and frozen tumor tissues in RLT buffer using the protocol provided in QIAGEN RNAeasy plus mini kit (#74034). RNA concentration and purity were measured using a NanoDrop spectrophotometer. cDNAs were synthesized from 1 μg of RNA using iScript cDNA synthesis kit (#1708891; Bio-Rad). 25 ng of total cDNA was used for qPCR with iTaq Universal SYBR Green Supermix (#172-5121; Bio-Rad). Samples were run in three technical replicates on a CFX96 real-time system (Bio-Rad). Assays consisted of three biological replicates. Primers used were as follow: msPTN (forward [fwd]: 5′-CTC​TGC​ACA​ATG​CTG​ACT​GTC-3′; reverse [rev]: 5′-CTT​TGA​CTC​CGC​TTG​AGG​CTT-3′), msCXCL5 (fwd: 5′-GCA​TTT​CTG​TTG​CTG​TTC​ACG​CTG-3′; rev: 5′-CCT​CCT​TCT​GGT​TTT​TCA​GTT​TAG​C-3′); msGAPDH (fwd: 5′-AGG​TCG​GTG​TGA​ACG​GAT​TTG-3′; and rev: 5′-TGT​AGA​CCA​TGT​AGT​TGA​GGT​CA-3′).

### mRNA sequencing and downstream analysis

Total RNA was isolated from tumors and cell lines from three biological replicates and shipped to Novogene Inc.. RNA integrity scores for samples were > 8. Sequencing was performed on an Illumina HiSeq 4000 sequencer (paired-end 150 nt) resulting in an average of 43 million reads, of which an average of 93% mapped to the mm10 reference genome. Fastq files were quality controlled with fastqc (https://www.bioinformatics.babraham.ac.uk/projects/fastqc/) and then aligned and assembled with the Hisat2/Stringtie pipeline (http://daehwankimlab.github.io/hisat2/; https://ccb.jhu.edu/software/stringtie/; Kim et al., 2019). Differential analysis was performed in R using Rsubread (https://bioconductor.org/packages/release/bioc/html/Rsubread.html; Liao et al., 2019) and edgeR https://bioconductor.org/packages/release/bioc/html/edgeR.html; Robinson et al., 2010). Statistical analysis was performed using the robust quasi-likelihood pipeline by edgeR. Metascape was used to perform Motif enrichment and TRRUST analysis on DEGs (Han et al., 2018). CIBERSORT analysis was performed at TIMER2.0 using the immune estimation feature. Heat map of neutrophil-associated genes was generated in GraphPad Prism.

### Statistics

All analyses were performed in GraphPad Prism 9. All mice used in this manuscript were females of similar age. All data is represented as mean ± SD. Two-tailed Student’s *t* test (paired or unpaired) and one-way ANOVA were used to compare two or more groups respectively. All experiments were performed in at least three biological replicates. A P value of <0.05 was considered significant.

### Online supplemental material

Fig. S1 validates elevated PTN expression in metastatic breast cancer cells and associates PTN expression with poor prognosis in breast cancer patients. Fig. S2 shows the expression of PTN in different cell types within human and mouse tumors. It also highlights PTN expression exclusively at the metastatic site in the lungs of tumor-bearing mice. Fig. S3 includes additional mouse validation experiments highlighting the benefit of PTN blockade in vivo. Fig. S4 provides supporting data for PTN knockdown validation as well as provides additional data on the in vitro effect of PTN on the NF-κB pathway in cancer cells. Fig. S5 validates Ly6G depletion in in vivo studies and provides a growth curve for in vivo experiments performed in Figs. 6 and 7.

## Supplementary Material

SourceData F5 contains original blots for Fig. 5.

SourceData FS1 contains original blots for Fig. S1.

SourceData FS4 contains original blots for Fig. S4.

## Data Availability

All raw data generated in this manuscript have been deposited in Gene Expression Omnibus under accession no. GSE200431.
