# Supplementary figures and images for: Pleiotrophin drives a prometastatic immune niche in breast cancer
Source: J Exp Med. 2023 Feb 24;220(5):e20220610. doi: 10.1084/jem.20220610 (PMC9998964; doi:10.1084/jem.20220610)

## Source file: Figure 5

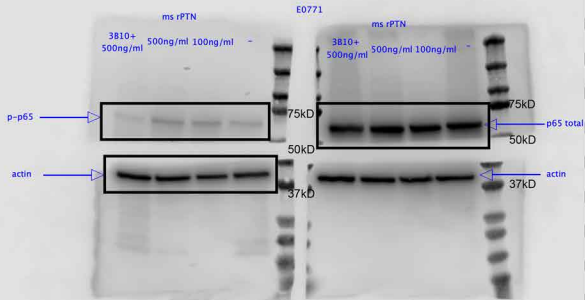

Supplement: SourceData F5 — contains original blots for Fig. 5. [file JEM_20220610_SourceDataF5.pdf]

Source file: Supplemental Figure 1

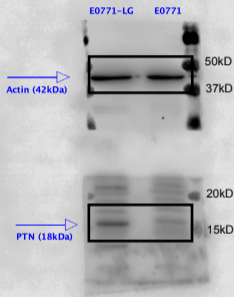

Supplement: SourceData FS1 — contains original blots for Fig. S1. [file JEM_20220610_SourceDataFS1.pdf]
