# Supplementary material for: Pleiotrophin drives a prometastatic immune niche in breast cancer
Source: J Exp Med. 2023 Feb 24;220(5):e20220610. doi: 10.1084/jem.20220610 (PMC9998964; doi:10.1084/jem.20220610)

Source file: Supplemental Figure 4C

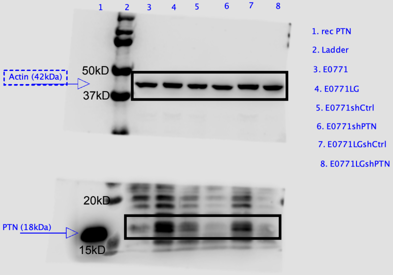

Source file: Supplemental Figure 4D

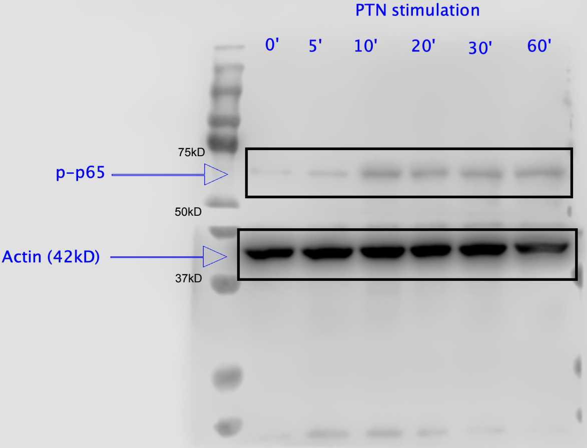

Source file: Supplemental Figure 4D

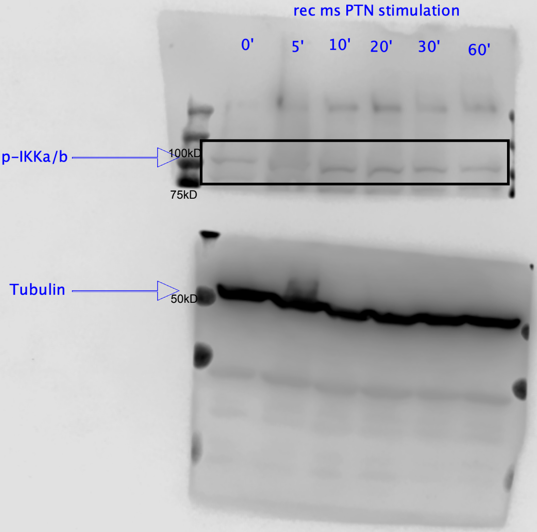

Source file: Supplemental Figure 4D

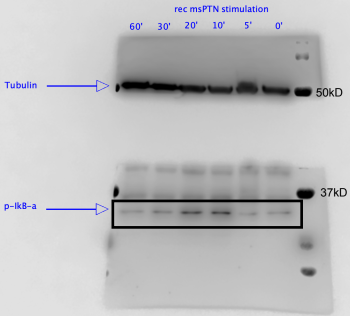

Source file: Supplemental Figure 4D

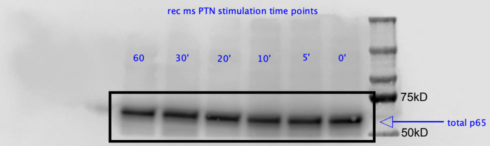

Supplement: SourceData FS4 — contains original blots for Fig. S4. [file JEM_20220610_SourceDataFS4.pdf]
